# The Impact of the Tumor Microenvironment on the Effect of IL-1β Blockade in NSCLC: Biomarker Analyses from CANOPY-1 and CANOPY-N Trials

**DOI:** 10.1158/2767-9764.CRC-24-0490

**Published:** 2025-04-18

**Authors:** Benjamin J. Solomon, Daniel S.W. Tan, Gilberto de Castro, Manuel Cobo, Marina Chiara Garassino, Jun Zhang, Bruce E. Johnson, Jay M. Lee, Pilar Garrido, Andrew A. Butler, Marc R. Pelletier, Alexander Savchenko, Lexiang Ji, Jan C. Brase, Rafael Caparica, David Demanse, Jincheng Wu, Claudia Bossen, Tony Mok

**Affiliations:** 1 Peter MacCallum Cancer Centre, Melbourne, Australia.; 2 National Cancer Centre Singapore, Duke-NUS Medical School, Singapore, Singapore.; 3 Instituto do Câncer do Estado de São Paulo, São Paulo, Brazil.; 4 Medical Oncology Intercenter Unit, Regional University Hospital and Virgen de la Victoria University Hospital, IBIMA, Málaga, Spain.; 5 Department of Medicine, Section Hematology Oncology, Thoracic Oncology, University of Chicago, Chicago, Illinois.; 6 Division of Medical Oncology, Department of Internal Medicine, University of Kansas Medical Center, Kansas City, Kansas.; 7 Dana-Farber Cancer Institute, Boston, Massachusetts.; 8 UCLA Health, Los Angeles, California.; 9 Hospital Universitario Ramón y Cajal, Madrid, Spain.; 10 Novartis Pharmaceuticals Corporation, East Hanover, New Jersey.; 11 Novartis BioMedical Research, Cambridge, Massachusetts.; 12 Precision Medicine, Novartis Pharmaceuticals Corporation, Cambridge, Massachusetts.; 13 Novartis Pharma AG, Basel, Switzerland.; 14 The Chinese University of Hong Kong, Hong Kong, China.

## Abstract

Preclinical studies have shown that interleukin (IL)-1β blockade can modulate the tumor microenvironment (TME) to activate antitumor immunity and, in combination with immune checkpoint inhibitors (ICIs), prevent cancer growth. Our study investigates if immune biomarkers in the TME affect outcomes in patients with non–small cell lung cancer (NSCLC) treated with the IL-1β inhibitor canakinumab plus an ICI-based therapy and describes canakinumab effects on the TMEs in these patients. Exploratory analyses were conducted in two prospective trials evaluating canakinumab combined with pembrolizumab-based regimens in patients with NSCLC: CANOPY-1 (first-line setting) and CANOPY-N (neoadjuvant setting). Immunohistochemistry (IHC) and transcriptomic analyses were performed on baseline tumor samples from CANOPY-1, and IHC and multiplex immunofluorescence analyses were performed on baseline and posttreatment tumor samples from CANOPY-N. Associations with clinical outcomes were evaluated. In CANOPY-1, in patients with low levels of T-cell infiltration in the tumor, the addition of canakinumab to a pembrolizumab-based regimen was associated with progression-free and overall survival improvements. Low levels of T-cell infiltration were associated with an immunosuppressive gene expression phenotype, supporting the role of low T-cell infiltration as a surrogate of an overall immunosuppressive TME. In CANOPY-N, a reduction in immunosuppressive cells in the TME was observed following canakinumab and pembrolizumab treatment. Our exploratory biomarker analyses from the CANOPY-1 and CANOPY-N trials suggest that IL-1β blockade may shift the TME toward an immune-activated status and that patients with immunosuppressive TME features could benefit from the addition of canakinumab to an ICI-based treatment.

**Significance:**

Patients with NSCLC with immunosuppressive tumor features and low T-cell infiltration derive less benefit from ICI-based treatment. Biomarker analyses presented here suggest that these patients may benefit from the addition of anti–IL-1β therapy to their treatment.

## Introduction

Lung cancer is the leading cause of cancer-related death globally (1, 2). The most frequent histologic subtype of lung cancer is non–small cell lung cancer (NSCLC), which accounts for about 85% of all cases (3). Immune checkpoint inhibitors (ICIs) have revolutionized NSCLC treatment by yielding an improvement in overall survival (OS), though only a subset of patients with NSCLC derive benefit from these agents (4, 5). In recent years, attempts have been made to identify predictive biomarkers of response to ICIs by characterizing the frequency and spatial distribution of immune cell subpopulations within the tumor stroma, which have been shown to affect the activity of ICIs (5). Studies have shown that increased cluster of differentiation (CD)8^+^ cytotoxic T-cell levels in tumor samples are associated with increased efficacy of ICIs, either as single agents or in combinations with other anticancer therapies, including in patients with NSCLC (6–9). In addition, immunophenotype categorization according to the distribution of T cells in the tumor stroma also predicts response to ICIs, as patients with an immune-inflamed phenotype (defined by high CD8^+^ T-cell infiltration) typically derive the most benefit from immunotherapy (10, 11).

The tumor microenvironment (TME) plays a key role in determining the tumor immunophenotype and is regulated by multiple cytokines. Interleukin (IL)-1β is a proinflammatory cytokine and a key mediator of the inflammatory response to infection and tissue injury (12). High levels of IL-1β have been linked to the promotion of lung cancer in preclinical models and associated with poor prognosis in patients with NSCLC (13, 14). In further preclinical models, IL-1β blockade has shown potential to interrupt tumor-promoting inflammation, modulate the TME toward an immune-activated status, and synergize with ICIs (15, 16).

The rationale for the CANOPY clinical trial program was based on preclinical data showing IL-1β as a key mediator of tumor-associated inflammation that promotes the development of NSCLC, together with the observed data from the Canakinumab Anti-inflammatory Thrombosis Outcome Study (CANTOS) looking at cardiovascular events, in which *post hoc* analyses showed that the use of canakinumab was associated with a dose-dependent reduction in lung cancer incidence and mortality (17). The CANOPY program consisted of four randomized trials that evaluated canakinumab in patients with NSCLC (18–21). Among these trials, two contained treatment arms with combined canakinumab and ICI therapy (CANOPY-1 and CANOPY-N; refs. 18, 21). CANOPY-1 evaluated the efficacy of pembrolizumab plus platinum-based chemotherapy combined with either canakinumab or placebo as first-line therapy for patients with advanced NSCLC. No progression-free survival (PFS) or OS benefit with the addition of canakinumab was observed in the overall study population or in the preplanned subgroups defined by C-reactive protein, IL-6, programmed death-ligand 1 (PD-L1), and tumor mutational burden status (18). CANOPY-N evaluated the efficacy of canakinumab or pembrolizumab, alone or in combination, as neoadjuvant treatment in patients with resectable NSCLC. No increase in the major pathologic response (MPR) rate was observed at surgery with the addition of canakinumab to pembrolizumab (21).

Despite their negative outcomes, the CANOPY-1 and CANOPY-N trials provide a unique opportunity to investigate the TME in the context of IL-1β blockade, with samples available from patients treated with an ICI with or without canakinumab and with corresponding patient outcome data available. Furthermore, in CANOPY-N, the effects of canakinumab with or without ICIs on the TME can be evaluated, with the availability of posttreatment tumor samples collected at surgery. In this context, the present article reports biomarker analyses and associations with clinical outcomes from patients enrolled in the CANOPY-1 and CANOPY-N trials.

## Materials and Methods

### Study designs

CANOPY-1 (NCT03631199) was a phase III, randomized, double-blind, global study evaluating the efficacy and safety of pembrolizumab plus platinum-based doublet chemotherapy combined with either canakinumab or placebo as first-line therapy for patients with advanced (stage IIIB–IV) NSCLC (Supplementary Fig. S1A). Further details are available in the original publication (18).

CANOPY-N (NCT03968419) was a phase II, randomized, open-label study evaluating the efficacy and safety of canakinumab or pembrolizumab, alone or in combination, as neoadjuvant treatment in patients with stage IB–IIIA NSCLC (Supplementary Fig. S1B). Patients were treated for a maximum of 6 weeks (two cycles), after which they were allocated to surgery according to local practice. Adjuvant treatment could be administered at the investigator’s judgment (21).

### Ethical oversight

Both studies were conducted in accordance with the Declaration of Helsinki and were performed in compliance with Good Clinical Practice guidelines. Both protocols and all amendments were reviewed and approved by an independent ethics committee or institutional review board at each site. All patients enrolled in the two studies provided written informed consent prior to study initiation and for the analysis of samples taken.

### Tissue sampling

For the CANOPY-1 trial, collection of archival or newly obtained baseline tumor tissue samples during screening was mandatory.

For the CANOPY-N trial, mandatory tumor tissue samples were collected at screening and at surgery.

### Translational analyses

#### IHC

In the CANOPY-1 trial, planned exploratory analyses included CD8^+^ T-cell infiltration.

In the CANOPY-N trial, planned exploratory analyses included the baseline level of and on-treatment change in total CD8 count, as well as cell populations defined by multiplex immunohistochemistry (IHC) and their relation to the percentage of viable tumor cells in the tumor specimens. The percentage of residual viable tumor cells and the percentages of stromal and necrotic components were determined (21).

PD-L1 expression was assessed using the IHC 22C3 pharmDx assay (18). The fraction of viable tumor cells (tumor proportion score; calculated as a percentage) that expressed PD-L1 was scored.

To assess the level of CD8^+^ immune cells (lymphocytes), a specific duplex chromogenic IHC assay comprising an anti-CD8 antibody (SP239, Abcam; RRID: AB_2756374) and an anti-panCK antibody (AE1/AE3/PCK26, Ventana: Roche Diagnostics; RRID: AB_ 2941938) was performed on Ventana Discovery Ultra at CellCarta (RRID: SCR_021254), and quantitative assessment was carried out using VisioPharm software at CellCarta (RRID: SCR_021711; ref. 19). Data were validated using a specific image algorithm to define the composition of CD8^+^ cells within different tumor compartments, including the tumor invasive margin (Supplementary Fig. S2). An algorithm was established under pathology review using panCK as the tumor-markedup area (or carcinoma). Semi-automatic scoring of CD8 in panCK-negative tumor stroma and the panCK-positive carcinoma cell compartment was performed to define the relative area/cell density inside/outside the panCK-positive tumor compartment, the stroma:tumor ratio, and the shape/size of tumor cell strands (Supplementary Fig. S2).

Specific cutoffs were developed and validated to classify tumor samples into three T-cell phenotypes based on the CD8/panCK IHC assay using the VisioPharm platform: inflamed phenotype, excluded phenotype, and desert phenotype. If the percentage of CD8 in the carcinoma central tumor was above 1.3%, it was classified as having an inflamed phenotype; if the CD8 percentage in the carcinoma central tumor was equal to or less than 1.3% and the CD8 percentage in the stroma central tumor was four-fold higher than the CD8 percentage in the carcinoma central tumor (CD8 stroma:tumor ratio >4), it was classified as having an excluded phenotype. Otherwise, it was classified as having a desert phenotype. The cutoff of 1.3% for the CD8 carcinoma and the stroma:tumor ratio was semi-quantitatively defined by pathologist review of IHC images and distributions of these two metrics to gate the patient subgroups with clear high CD8^+^ infiltration (inflamed) and a high stroma:tumor ratio (excluded). The cutoff of 1.3% represented the highest quartile of the CD8 IHC percentage in the carcinoma region, and the stroma:tumor ratio cutoff of >4 was determined by pathologist review of the distribution. Samples with no eligible read-outs for CD8 percentage or stroma:tumor ratio were classified as not available (NA). All CD8 IHC results generated in the CANOPY-1 trial were used to define the CD8^+^ phenotype classification, and the cutoffs and subgroups were locked down and documented prior to unblinding the trial. Representative pictures of the phenotypes are shown in Supplementary Fig. S2. For samples with paired RNA sequencing (RNA-seq) data (Supplementary Fig. S3A), T-cell–inflamed gene signature (Supplementary Fig. S3B) and fibroblast gene signatures (Supplementary Fig. S3C; ref. 22) were respectively enriched in inflamed and excluded T-cell phenotypes, further validating the phenotype classification.

### Quantitative immunofluorescence staining, imaging, and analysis

Formalin-fixed, paraffin-embedded tissue samples were stained on the Bond RX automated stainer (Leica Biosystems). Staining steps were performed at room temperature. Endogenous peroxidase was blocked using Peroxidazed 1 (Biocare Medical), followed by incubation with a protein-blocking solution (1× antibody diluent per block, Akoya Biosciences) to reduce nonspecific antibody staining. For the CD3, CD11b, CD19, CD66b, CD163, and CD56 assay, slides were then sequentially stained with each of the following antibodies for 1 hour: anti-CD3 (LN10 RTU, Leica Biosystems; RRID: AB_3073619), 0.56 μg/mL anti-CD11b (EP1345Y, Abcam; RRID: AB_868788), 0.175 µg/mL anti-CD19 (BT51E, Leica Biosystems; RRID: AB_10555427), 1.0 μg/mL anti-CD66b (G10F5, BD Biosciences; RRID: AB_396066), anti-CD163 (10D6 RTU, Leica Biosystems; RRID: AB_2756375), and 0.0985 µg/mL anti-CD56 (MRQ-42, Millipore Sigma; RRID: AB_2941091). EnVision+ HRP Mouse or Rabbit (Agilent; RRID: AB_2827819 or AB_2630375) secondaries were used for 30 minutes, and Opal 480, Opal 520, Opal 570, Opal 620, Opal 690, and Opal 780 (Akoya Biosciences) detection reagents were used to visualize each antibody. For the CD3, FOXP3, granzyme B, and cytokeratin assays, slides were sequentially stained with each of the following antibodies for 1 hour: anti-CD3 (LN10 RTU, Leica Biosystems; RRID: AB_3073619), 2.5 μg/mL anti-FOXP3 (D2W8E, Cell Signaling Technology; RRID: AB_2747370), 2.0 μg/mL anti-granzyme B (D6E9W, Cell Signaling Technology; RRID: AB_2799313), and 0.427 μg/mL anti-cytokeratin (AE1/AE3, Agilent; RRID: AB_2132885). EnVision+ HRP Mouse or Rabbit (Agilent; RRID: AB_2827819 or AB_2630375) secondaries were used for 30 minutes, and Opal 520, Opal 570, Opal 620, and Opal 690 (Akoya Biosciences) detection reagents were used to visualize each antibody. Finally, slides were stained with Spectral DAPI (Akoya Biosciences) for 10 minutes prior to coverslipping with ProLong Gold (Thermo Fisher Scientific). Whole-slide scan fluorescence images were acquired on the PhenoImager HT (Akoya Biosciences) at 0.5 μm/pixel (20×) image resolution. Slides were spectrally unmixed using inForm v2.4.5 (Akoya Biosciences; RRID: SCR_019155), before analysis using AQUA technology v3.2.5.0 (Navigate BioPharma Services), as described previously by Johnson and colleagues (23). Representative pictures of phenotypes of interest are shown as CD11b/CD66b stained for polymorphonuclear granulocytes (or neutrophils, Supplementary Fig. S4A), CD163 stained for monocyte-lineage macrophages [or tumor-associated macrophages (TAM); Supplementary Fig. S4B], and FOXP3/CD3 stained for regulatory T cells (Supplementary Fig. S4C).

### RNA-seq

Ribosomal (r)RNA from extracted total RNA was depleted using RNase H. The rRNA-depleted sample was then fragmented, converted to complementary DNA, and carried through the remaining steps of next-generation sequencing library construction, end repair, A-tailing, indexed adapter ligation, and polymerase chain reaction amplification, using the TruSeq RNA v.2 Library Preparation Kit (Illumina). The captured library was pooled with other libraries, each having a unique adapter index sequence, and applied to a sequencing flow cell. The flow cell underwent cluster amplification and massively parallel sequencing by synthesis using Illumina v.4 chemistry and paired-end 100-bp reads (Illumina).

Sequence data were aligned to the reference human genome (build hg19) using STAR v.2.4.0e (RRID: SCR_004463; ref. 24). Mapped reads were then used to quantify transcripts with HTSeq v.0.6.1p1 (RRID: SCR_005514; ref. 25). Gene expression data were normalized using the trimmed mean of *M*-value normalization, as implemented in the edgeR R/Bioconductor package v.3.20.9 (RRID: SCR_012802; ref. 26).

Pathway/gene set expression score was derived using the arithmetic mean of all gene expression (trimmed mean of *M* values corrected log_2__cpm) in each set. To quantify T-cell–inflamed score from RNA-seq, an 18-gene signature (27) was used to define T-cell–high and T-cell–low subgroups based on the median cutoff.

### Statistical analysis

Data were summarized using descriptive statistics. Categorical data were presented as frequencies and percentages. PFS and OS were presented descriptively using the Kaplan–Meier method and were summarized by presenting the median time to event along with the corresponding 95% confidence interval (CI). Cox proportional hazards regression models were used to investigate the prognostic and predictive roles of biomarkers for PFS and OS and to estimate hazard ratios (HRs) and 95% CIs. Stratification factors [PD-L1 expression (<1% vs. ≥1%), histology (squamous vs. nonsquamous), and geographical region (East Asia vs. North America and Western Europe vs. rest of the world)] were all included in the models, consistent with the clinical statistical analysis method (18).

For TME principal component analysis (PCA), in-house and published gene sets were used as inputs for PCA (Supplementary Table S3), and IHC features, including CD8 percentages in carcinoma, stroma, and total regions, and PD-L1 tumor proportion score percentage were also included as supplemental variables. Principal components were used to understand the dimension of variability of the TME features.

For TME linear regression-based analysis, signatures were further extended to include additional collection of TME signatures derived based on single-cell RNA-seq analysis (28); the TME signature score by CD8 subgroup was calculated as the marginal mean after adjusting total ESTIMATE immune score using linear regression model (signature of interest ∼ ESTIMATE score + CD8 subgroup), and ESTIMATE immune score was calculated based on the immune signature (29).

### Primary endpoints

The primary endpoints of CANOPY-1 were PFS, defined as the time from randomization to disease progression or death due to any cause, and OS, defined as the time from randomization to death due to any cause (18). All results are based on clinical database lock following the final OS cutoff date of August 9, 2021, except for PFS analysis results, which are based on the primary PFS cutoff date of May 18, 2020.

The primary endpoint for efficacy in CANOPY-N was MPR, defined as ≤10% residual viable tumor cells in primary resected tumor tissue. MPR was established to assess the amount of residual viable tumor cells present in the original tumor bed using hematoxylin and eosin-stained slides. The methodology was validated at the partner Clinical Laboratory Improvement Amendments laboratory following International Association for the Study of Lung Cancer published recommendations for the pathologic assessment of lung cancer resection specimens after neoadjuvant therapy (21, 30). All results are based on clinical database lock following the final clinical cutoff date of August 15, 2022.

### Data availability

Novartis is committed to sharing data and supporting clinical documents from eligible studies. Trial data availability can be found on www.clinicalstudydatarequest.com. In cases where the data are not yet available, they can be requested through this website.

The authors declare that all relevant aggregate biomarker data supporting the findings of this study are available within the article and its supplementary information files. In accordance with the Health Insurance Portability and Accountability Act, we do not have institutional review board approval or patient consent to share individualized patient genomic data, so data cannot be reported in a public data repository. The data generated in this study are available upon request from the corresponding author.

## Results

### Patient characteristics

In CANOPY-1, of the total 643 patients enrolled, 500 had evaluable CD8 IHC results and 298 patients had whole-transcriptome analysis data available (Supplementary Table S1). No imbalances were observed in the baseline characteristics when comparing the subset of patients with biomarker results available against the full analysis set population. In addition, no imbalances between the treatment groups in terms of baseline characteristics were observed in the subgroups defined by CD8 level and T-cell phenotype (Table 1).

**Table 1:** Baseline characteristics for CANOPY-1 and CANOPY-N population subsets

	CANOPY-1											CANOPY-N			
	CD8 level (median cutoff)				CD8 phenotype						All patients	Treatment arm			All patients
	CD8 low (*n* = 250)		CD8 high (*n* = 250)		Desert (*n* = 81)		Excluded (*n* = 290)		Inflamed (*n* = 128)			Can	Can + pembro	Pembro	
											(*N* = 643)	(*n* = 35)	(*n* = 35)	(*n* = 18)	(*N* = 88)
	Placebo	Can	Placebo	Can	Placebo	Can	Placebo	Can	Placebo	Can					
Age, *n* (%)															
<65 years	61 (53)	78 (58)	80 (58)	71 (63)	26 (65)	26 (63)	41 (59)	36 (61)	74 (52)	86 (59)	370 (58)	15 (43)	11 (31)	5 (28)	31 (35)
≥65 years	54 (47)	57 (42)	57 (42)	42 (37)	14 (35)	15 (37)	28 (41)	23 (39)	69 (48)	61 (41)	273 (42)	20 (57)	24 (69)	13 (72)	57 (65)
Sex, *n* (%)															
Female	32 (28)	40 (30)	35 (26)	32 (28)	12 (30)	11 (27)	19 (28)	17 (29)	36 (25)	44 (30)	184 (29)	13 (37)	14 (40)	9 (50)	36 (41)
Male	83 (72)	95 (70)	102 (74)	81 (72)	28 (70)	30 (73)	50 (72)	42 (71)	107 (75)	103 (70)	459 (71)	22 (63)	21 (60)	9 (50)	52 (59)
ECOG PS, *n* (%)															
0	46 (40)	52 (39)	51 (37)	46 (41)	15 (38)	11 (27)	27 (39)	19 (32)	55 (38)	68 (46)	248 (39)	22 (63)	19 (54)	13 (72)	54 (61)
1	69 (60)	83 (61)	86 (63)	67 (59)	25 (62)	30 (73)	42 (61)	40 (68)	88 (62)	79 (54)	395 (61)	13 (37)	16 (46)	5 (28)	34 (39)
Smoking history, *n* (%)															
Former/current smoker	93 (81)	104 (77)	121 (88)	94 (83)	33 (82)	33 (80)	63 (91)	48 (81)	118 (83)	117 (80)	520 (81)	32 (91)	29 (83)	15 (83)	76 (86)
Never smoker	22 (19)	31 (23)	16 (12)	19 (17)	7 (18)	8 (20)	6 (9)	11 (19)	25 (17)	30 (20)	123 (19)	3 (9)	6 (17)	3 (17)	12 (14)
Histology, *n* (%)															
Nonsquamous	77 (67)	94 (70)	95 (69)	76 (67)	31 (78)	27 (66)	48 (70)	37 (63)	93 (65)	105 (71)	448 (70)	20 (57)	21 (60)	11 (61)	52 (59)
Squamous	38 (33)	41 (30)	42 (31)	37 (33)	9 (22)	14 (34)	21 (30)	22 (37)	50 (35)	42 (29)	195 (30)	15 (43)	14 (40)	7 (39)	36 (41)

Abbreviations: Can, canakinumab; ECOG PS, Eastern Cooperative Oncology Group performance status; pembro, pembrolizumab.

Characteristics from patients in the CANOPY-N study are provided in Table 1. Seventy-three patients had evaluable CD8 IHC at screening and 67 at surgery, and up to 69 patients had evaluable multiplex immunofluorescence (IF) data at screening and 67 at surgery (depending on the multiplex IF panel; Supplementary Table S1).

### Prognostic impact of T-cell phenotype in CANOPY-1

**Figure 1:**
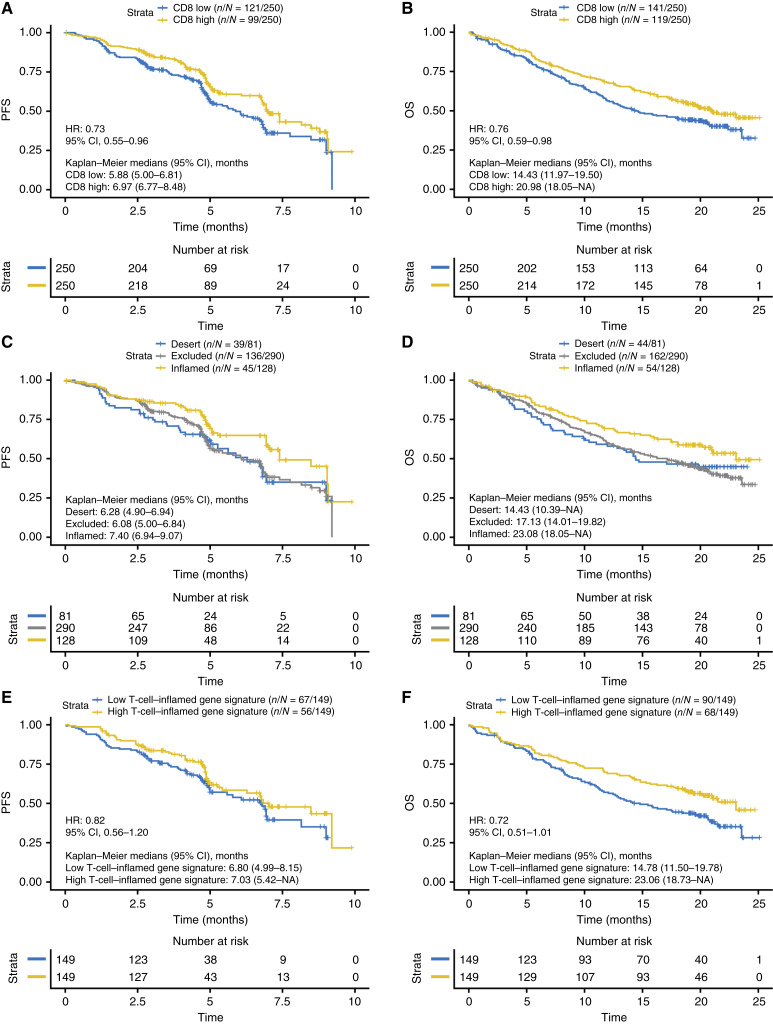
(**A**) PFS and (**B**) OS by CD8 IHC (low and high by median cutoff); (**C**) PFS and (**D**) OS by CD8 phenotype; and (**E**) PFS and (**F**) OS by T-cell–inflamed gene signature (low and high by median cutoff) in CANOPY-1.

In the CANOPY-1 trial, the prognostic impact of T-cell phenotype was first assessed in all patients, with both treatment arms pooled. Patients with high CD8^+^ T-cell (vs. low CD8^+^ T-cell) infiltration were defined using the median cutoff for total CD8^+^ T cells in all tumor samples. The median total number of CD8^+^ T cells was 2.47%. A high CD8^+^ T-cell infiltration was associated with improved PFS (HR, 0.73; 95% CI, 0.55–0.96; Fig. 1A). Similarly, the high CD8^+^ T-cell infiltration was associated with improved OS, in comparison to the low CD8^+^ T-cell infiltration subgroup (HR, 0.76; 95% CI, 0.59–0.98; Fig. 1B).

A similar trend was also observed when patients were classified by T-cell phenotype, with those classified as having inflamed tumors (*n* = 128) having both longer PFS (7.40 months; 95% CI, 6.94–9.07) and OS (23.08 months; 95% CI, 18.05 to not estimable) compared with the other two subgroups [desert (*n* = 81) and excluded (*n* = 290) phenotypes; Fig. 1C and D]. Of note, the patients with an immune desert T-cell phenotype were also predominantly in the CD8-low subgroup, whereas those with an inflamed T-cell phenotype were predominantly in the CD8-high subgroup. Patients with an excluded T-cell phenotype were categorized in both the CD8-low and CD8-high subgroups (Supplementary Fig. S5A and S5B).

Similar trends were also observed when evaluating patient outcomes per T-cell activation status, assessed with a T-cell gene expression signature (*n* = 298; ref. 27): patients with a high expression of T-cell–associated genes had numerically longer median PFS (HR, 0.82; 95% CI, 0.56–1.20; Fig. 1E) and longer median OS (HR, 0.72; 95% CI, 0.51–1.01; Fig. 1F) compared with those with a low score in this signature.

### Canakinumab benefit according to CD8^+^ T-cell infiltration and phenotype in CANOPY-1

To investigate if CD8^+^ T-cell infiltration predicted the benefit of adding canakinumab to pembrolizumab and platinum-based chemotherapy, exploratory analyses were conducted per treatment arm in CANOPY-1.

**Figure 2:**
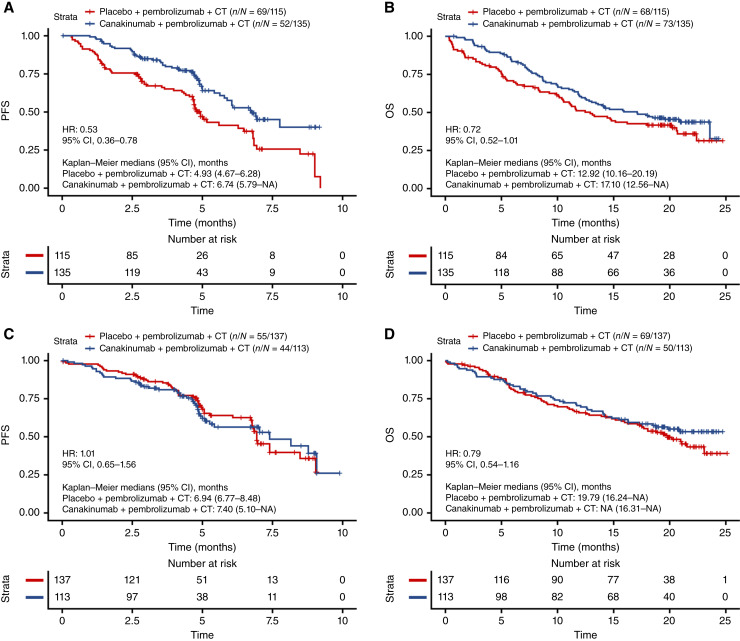
(**A**) PFS and (**B**) OS by treatment in CD8 low; (**C**) PFS and (**D**) OS by treatment in CD8 high for CANOPY-1. CT, chemotherapy.

Among patients with a low CD8 count (below median; *n* = 250), the addition of canakinumab to chemotherapy and immunotherapy was associated with longer PFS when compared with placebo (HR, 0.53; 95% CI, 0.36–0.78; Fig. 2A). A similar trend was also observed for OS (HR, 0.72; 95% CI, 0.52–1.01; Fig. 2B).

Among patients with a high CD8 count (above median; *n* = 250), no benefit in terms of PFS (HR, 1.01; 95% CI, 0.65–1.56; Fig. 2C) or OS (HR, 0.79; 95% CI, 0.54–1.16; Fig. 2D) was observed in the canakinumab arm compared with the placebo arm.

**Figure 3:**
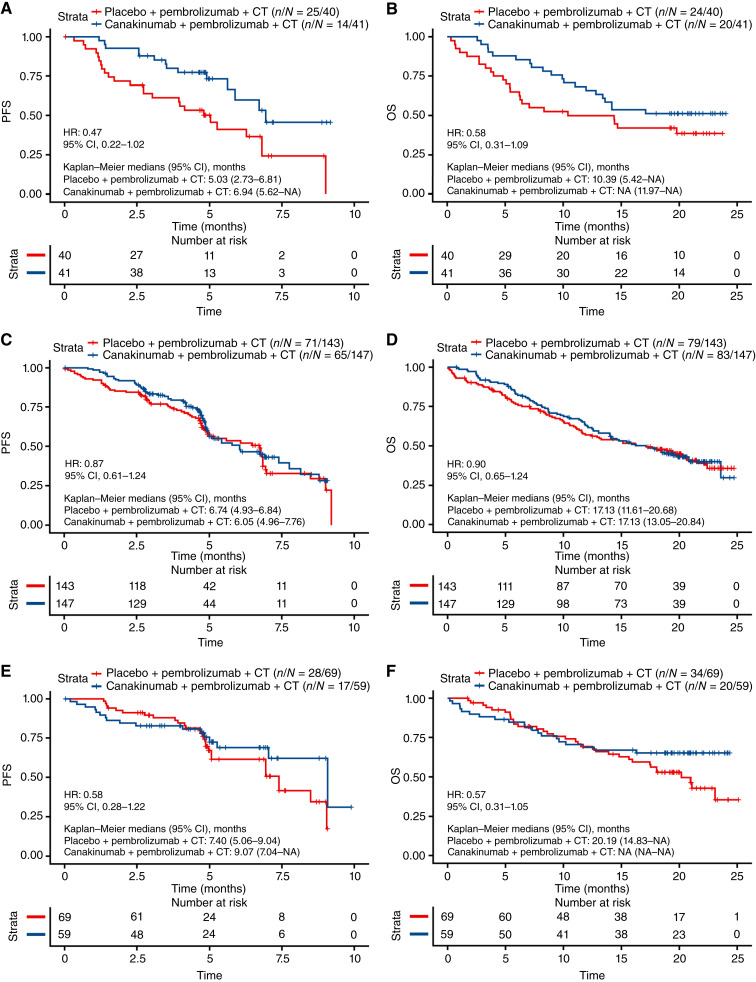
(**A**) PFS and (**B**) OS by treatment in CD8^+^ desert tumors; (**C**) PFS and (**D**) OS by treatment in CD8^+^ excluded tumors; and (**E**) PFS and (**F**) OS by treatment in CD8^+^-inflamed tumors subgroups in CANOPY-1. CT, chemotherapy.

Among patients whose tumors were classified as having a CD8^+^ desert T-cell phenotype (*n* = 81), trends toward increased PFS (HR, 0.47; 95% CI, 0.22–1.02; Fig. 3A) and OS (HR, 0.58; 95% CI, 0.31–1.09; Fig. 3B) were observed in patients treated with canakinumab compared with those who received placebo.

In patients with a CD8^+^ excluded T-cell phenotype (*n* = 290), no differences between canakinumab and placebo arms were observed in terms of PFS (HR, 0.87; 95% CI, 0.61–1.24; Fig. 3C) or OS (HR, 0.90; 95% CI, 0.65–1.24; Fig. 3D).

In patients with CD8^+^-inflamed tumors (*n* = 128), no significant benefit was observed in patients treated with canakinumab versus those treated with placebo in terms of PFS (HR, 0.58; 95% CI, 0.28–1.22; Fig. 3E) or OS (HR, 0.57; 95% CI, 0.31–1.05; Fig. 3F).

We further evaluated how CD8 subgroup and treatment arm were associated with clinical outcomes; however, the interactions were not statistically significant (Supplementary Table S2).

### Canakinumab benefit according to T-cell gene signature in CANOPY-1

In CANOPY-1, when assessing CD8^+^ T-cell infiltration using a T-cell–inflamed gene expression signature (*n* = 298; ref. 27), in those with a low T-cell–inflamed signature, prolonged PFS was observed among patients treated with canakinumab in combination with pembrolizumab and chemotherapy (HR, 0.53; 95% CI, 0.31–0.90; Supplementary Fig. S6A). Although no significant association was observed in terms of OS (HR, 0.77; 95% CI, 0.49–1.19; Supplementary Fig. S6B), the median OS was numerically higher among patients who received canakinumab. No difference in outcome was observed with canakinumab treatment in patients with a high T-cell–inflamed gene signature (Supplementary Fig. S6C and S6D). Overall, these findings suggest that patients with low levels of CD8^+^ T-cell infiltration and/or low T-cell activity in their tumors may benefit from the addition of canakinumab to standard of care.

### Canakinumab benefit according to CD8^+^ T-cell subgroup and PD-L1 level in CANOPY-1

In the CANOPY-1 trial subgroup analyses, canakinumab did not show benefit when compared with placebo in any of the predefined PD-L1 subgroups (18). In the present analyses, we observed an enrichment of PD-L1–negative tumors among patients with low levels of CD8^+^ T-cell infiltration (Supplementary Fig. S7A and S7B) in CANOPY-1. Further analyses were conducted in CD8 and PD-L1 subgroups to assess the impact of PD-L1 level on response to canakinumab in the CD8 subgroups.

**Figure 4:**
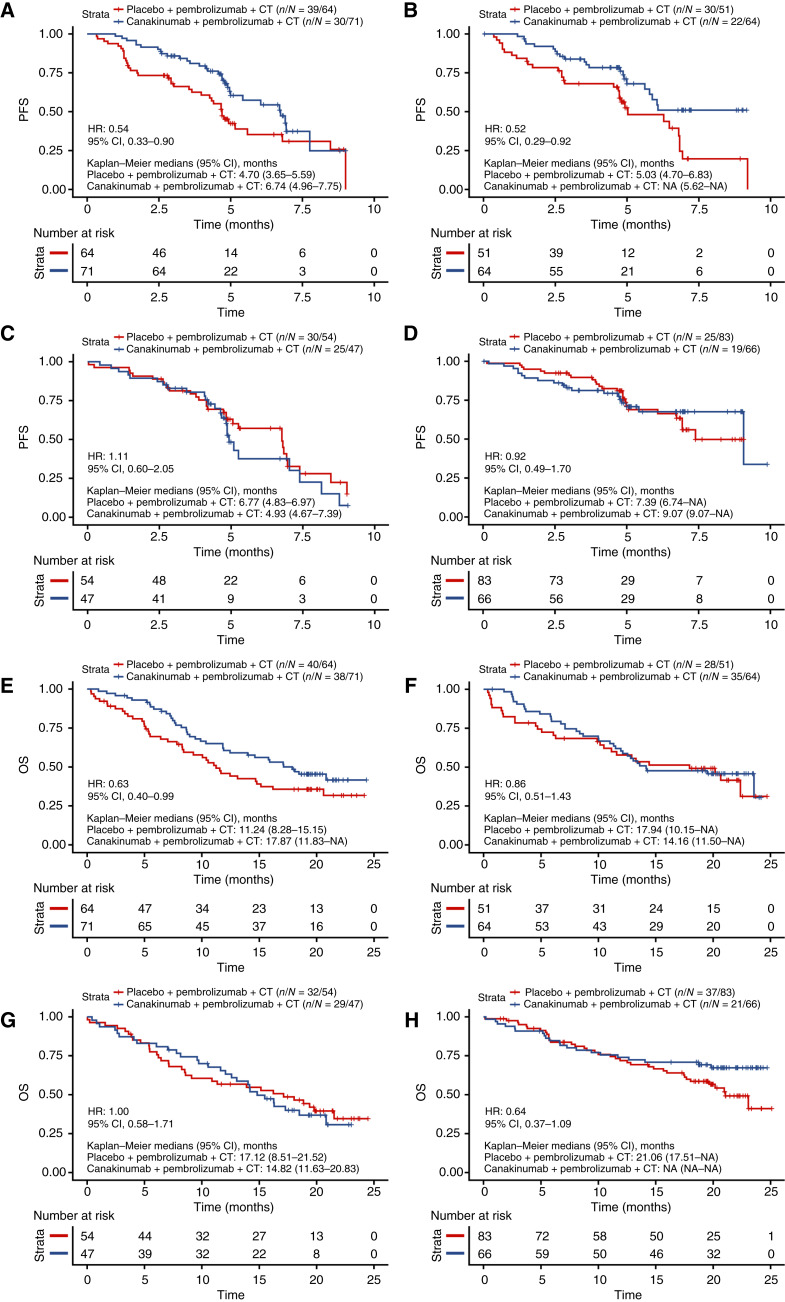
PFS by treatment in: (**A**) CD8 low and PD-L1 <1%; (**B**) CD8 low and PD-L1 ≥1%; (**C**) CD8 high and PD-L1 <1%; (**D**) CD8 high and PD-L1 ≥1%. OS by treatment in: (**E**) CD8 low and PD-L1 <1%; (**F**) CD8 low and PD-L1 ≥1%; (**G**) CD8 high and PD-L1 <1%; (**H**) CD8 high and PD-L1 ≥1% subgroups for CANOPY-1. CT, chemotherapy.

PD-L1 expression did not add predictive value for any association between PFS and canakinumab treatment, and both PD-L1–negative and PD-L1–positive tumors were associated with longer PFS in the CD8-low subgroup with canakinumab treatment, whereas both PD-L1–negative and PD-L1–positive tumors showed no association between PFS and canakinumab in the CD8-high subgroup (Fig. 4A–D).

Noteworthy, among patients with PD-L1–negative tumors (<1%) and low CD8 (below median), a longer OS was observed when canakinumab was added to pembrolizumab and chemotherapy (*n* = 135; HR, 0.63; 95% CI, 0.40–0.99; Fig. 4E), whereas no OS difference between canakinumab and placebo was observed in patients with PD-L1–positive tumors (≥1%) and low CD8 (*n* = 115; HR, 0.86; 95% CI, 0.51–1.43).

In patients with PD-L1–negative tumors and high CD8 (*n* = 101; HR, 1.00; 95% CI, 0.58–1.71) or with PD-L1–positive tumors and high CD8 (*n* = 149; HR, 0.64; 95% CI, 0.37–1.09), no OS difference between canakinumab and placebo was observed (Fig. 4F–H).

### TME analysis in CD8 subgroups in CANOPY-1

**Figure 5:**
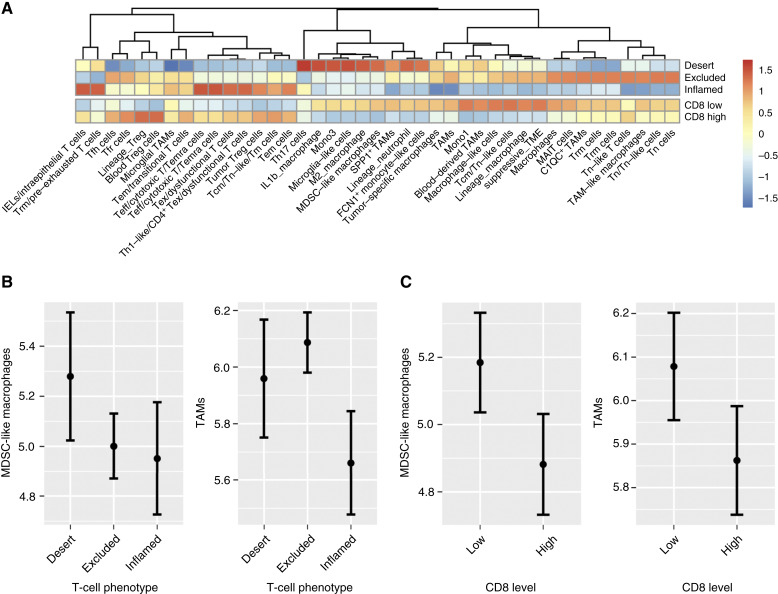
Relative gene signatures by CD8 subgroup in CANOPY-1. **A,** Heatmap showing enrichment of the TME gene signatures in the CD8 subgroups. **B,** MDSC-like macrophage and TAM gene signatures by T-cell phenotype and (**C**) and in high and low CD8 subgroups. The signature score was calculated as estimated marginal means after adjustment of total immune level (ESTIMATE immune score). Signature source (28). FCN1, ficolin-1; IEL, intraepithelial lymphocytes; MAIT, mucosal-associated invariant T cells; SPP1, phosphoprotein 1; Teff, effector T cell; Treg, regulatory T cell.

To further study the TME in patients with low CD8^+^ T-cell infiltration, we leveraged our transcriptomic data with two different approaches. First, we used a targeted approach investigating a curated list of gene signatures (cell types of interest) and their enrichment depending on CD8^+^ T-cell infiltration. As expected, T-cell gene signatures were enriched in tumor samples classified as CD8 high (above the median) or with an inflamed T-cell phenotype (Fig. 5A). On the other hand, tumors classified as CD8 low (below the median) or with a desert T-cell phenotype generally were enriched for suppressive myeloid TME features (Fig. 5A); for example, myeloid-derived suppressor cell (MDSC)-like macrophage and TAM gene signatures were enriched in CD8 low and desert T-cell phenotype samples (Fig. 5B and C).

Second, we used a PCA with all the gene signatures and IHC features (CD8 and PD-L1) to deconvolve independent TME features. In the PCA, the first two dimensions represented total immune infiltration (dimension 1, 44% of variability) and balance between immunosuppressive signatures and antitumor immunity (dimension 2, 19% of variability; Supplementary Fig. S8). Tumors classified as having low CD8^+^ T-cell infiltration (below the median) had lower total immune infiltration (Supplementary Fig. S9A) and showed a shift toward an immunosuppressive signature (Supplementary Fig. S9B) compared with tumors classified as having high CD8^+^ T-cell infiltration.

### Canakinumab effects on the TME in CANOPY-N

The neoadjuvant CANOPY-N trial, with paired tissue sample collection at baseline (before treatment) and at surgery (after treatment), provided an opportunity to study the impact of canakinumab on the TME. In addition to CD8^+^ T cells measured by IHC, additional cell types were detected by multiplex IF.

**Figure 6:**
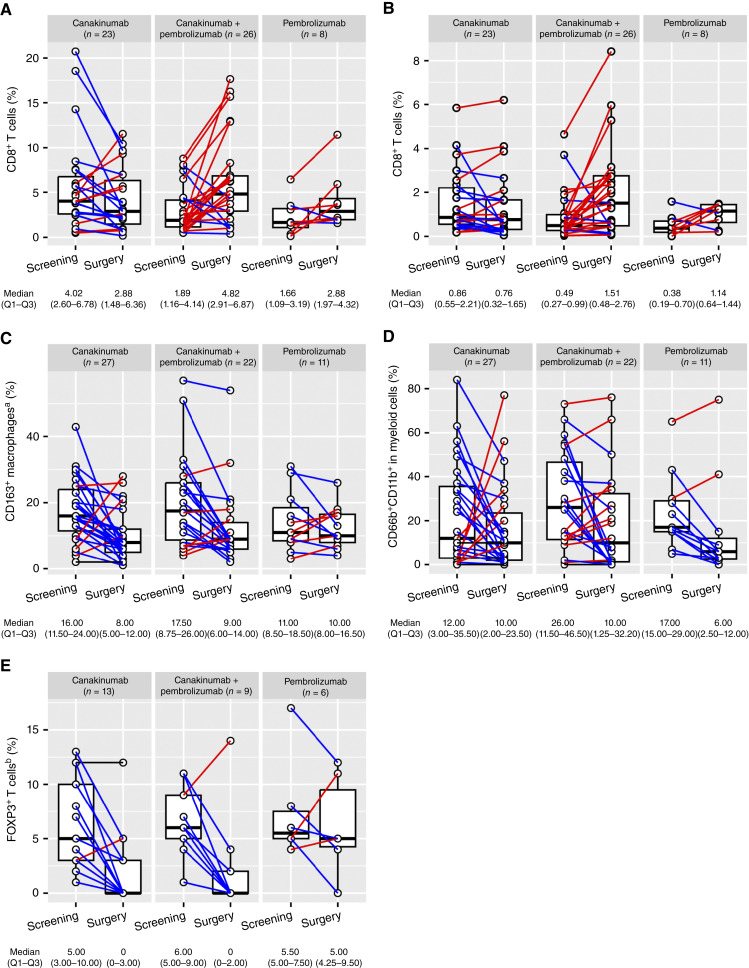
(**A**) Total CD8^+^ T cells, (**B**) intratumoral CD8^+^ T cells, (**C**) CD163^+^ TAMs, (**D**) CD66b^+^CD11b^+^ myeloid cells, (**E**) and FOXP3^+^ regulatory T cells by treatment arm in CANOPY-N. ^a^CD163 is a marker for TAMs and was normalized by total detectable cells. ^b^FOXP3 is a marker for regulatory T cells and was normalized by the abundance of CD3^+^ cells. CD66b expression is a marker for granulocytes including neutrophils, and CD11b is a marker for myeloid cells.

In patients who received pembrolizumab (alone or combined with canakinumab), an increase in the levels of intratumoral CD8^+^ T cells was observed. This effect did not occur in patients who received canakinumab alone. The increase in CD8^+^ T-cell infiltration was more pronounced in patients who received the canakinumab plus pembrolizumab combination, for both total and intratumoral CD8^+^ T cells; however, due to the small size of the cohort, in particular in the pembrolizumab arm, further data would be needed to validate this potential synergistic effect (Fig. 6A and B).

Changes in T-cell phenotypes were also assessed from baseline to surgery. There was an increase, both numerically (Supplementary Fig. S10A) and as a percentage of patients (Supplementary Fig. S10B), in tumor samples categorized as having an inflamed T-cell phenotype in the pembrolizumab-containing arms, most pronounced in the canakinumab plus pembrolizumab arm.

These results are in line with the MPRs observed in the different arms reported for the study at final analysis, with MPR rates of 2.9%, 17.1%, and 16.7% in the canakinumab, canakinumab + pembrolizumab, and pembrolizumab arms, respectively. In addition, when considering the percentages of viable tumor cells in surgical samples in all arms, mostly pembrolizumab-containing arms showed tumors with a low percentage (<20%) of viable tumor cells (Supplementary Fig. S11A; 9 patients of 33 with available viable tumor cells in the pembrolizumab + canakinumab arm and 4 patients of 17 in the pembrolizumab arm, with the exception of 1 patient of 31 in the canakinumab arm; ref. 21).

To determine if patients whose tumors had low T-cell infiltration at baseline may also benefit from canakinumab, as observed in CANOPY-1, the percentage of viable tumor cells in different arms was explored in patients with low CD8^+^ T-cell infiltration. In patients with low CD8^+^ T-cell infiltration at baseline (CD8 low), more patients had a low percentage of viable tumor cells (<20%) in the combination arm (6 of 19 patients with low baseline CD8^+^ T-cell infiltration achieving a low percentage of viable tumor cells) compared with patients with high CD8^+^ T-cell infiltration at baseline (2 of 11 patients). No patients with low CD8^+^ T-cell levels in the pembrolizumab-only arm presented with a low percentage of viable tumor cells (Supplementary Fig. S11B and S11C).

The impacts of canakinumab on other immune cell types within the TME were evaluated by multiplex IF assays. In the myeloid compartment, canakinumab treatment was associated with a reduction in CD163^+^ cells in the TME (Fig. 6C). The reduction was observed both with canakinumab alone and with canakinumab in combination with pembrolizumab, whereas pembrolizumab alone did not modulate CD163^+^ cells, supporting a canakinumab-mediated effect (Fig. 6C). A decrease in CD66b^+^CD11b^+^ granulocytic cells was observed, being more prominent in arms containing pembrolizumab compared with canakinumab monotherapy (Fig. 6D). Beyond myeloid cells, reductions in FOXP3^+^ cells (as a proportion of total CD3 cells) were observed in canakinumab arms but not in the pembrolizumab arm (Fig. 6E).

## Discussion

Although the CANOPY studies did not meet their primary endpoints (PFS/OS in CANOPY-1; MPR in CANOPY-N), there is a strong preclinical and clinical rationale for targeting IL-1β in lung cancer. IL-1β blockade has the potential to interrupt tumor-promoting inflammation, shift the tumor immune microenvironment toward an immune-activated status, and synergize with ICIs (15). The exploratory analyses performed in CANOPY-1 and CANOPY-N provide insights into the TME features in patients who received IL-1β blockade, which could help identify patients with NSCLC who may benefit from the addition of IL-1β inhibition to an ICI.

Using CANOPY-1 tissue samples, three correlative analyses on CD8^+^ T-cell subgroups were performed to understand the role of baseline T cells in response to ICI-based treatment, namely, total CD8 count in tumor samples (low vs. high), T-cell phenotype (immune desert vs. excluded vs. inflamed), and inflamed T-cell gene expression signature (low vs. high). All three analyses are in line with prior studies that showed high CD8^+^ T-cell infiltration within the TME is associated with better prognosis in patients with NSCLC who received an ICI-based treatment (6). Noteworthy, an artificial intelligence-based algorithm (Lunit SCOPE IO) that defined T-cell phenotypes in NSCLC also demonstrated that patients whose tumors had immune desert and immune excluded phenotypes had the least benefit from ICI monotherapy (31). Taken together, these data show that patients with NSCLC whose tumors have low CD8^+^ T-cell infiltration derive less benefit from current standard ICI-based treatment; therefore, additional treatment strategies are needed to improve outcomes in this population (6, 32).

CD8^+^ T-cell infiltration and localization, which are important for anticancer immunity, are modulated by components of the TME that may promote either an immunosuppressive or immune-active environment. For example, the presence of cytokines such as TGF-β, or cells such as cancer-associated fibroblasts and TAMs in the TME can inhibit the functioning and trafficking of T cells (33–35). Conversely, high levels of immunogenic cytokines, such as IL-2 and IL-15, stimulate the recruitment of immune effector cells, such as CD8^+^ and natural killer cells, to create an immune-active TME that facilitates the antitumor activity of ICIs (6, 36). The transcriptomic analyses of baseline tissue samples from patients in CANOPY-1 showed the gene expression profile of these tumors varies according to their T-cell phenotype classification. Immune desert tumors showed an enrichment of gene signatures from myeloid-derived cells associated with immunosuppression (M2 macrophages, MDSC-like macrophages, and neutrophils), whereas gene signatures from effector T cells were enriched among T-cell–inflamed tumors. An analysis of the transcriptomic data, using PCA, further revealed that patients with low T-cell infiltration had a shift toward expressing immunosuppressive gene signatures, shifting the balance toward immunosuppression. These results suggest that low CD8^+^ T-cell infiltration is associated with further immunosuppressive features, such as an enrichment of myeloid cells and an increased expression of genes that inhibit immune activation in the TMEs of these tumors. In this context, our findings suggest that low levels of infiltrating CD8^+^ T cells are surrogates of an overall immunosuppressed TME.

In preclinical models of NSCLC, IL-1β blockade exerts an immunogenic effect in the TME via the recruitment and activation of immune effector CD8^+^ T cells, while reducing peritumoral infiltration by immunosuppressive cells such as MDSCs (15, 37, 38). Single-cell RNA-seq, profiling of solid tumor samples, including NSCLC, pancreatic ductal adenocarcinoma, and microsatellite stable colorectal cancer samples, has shown that the IL-1β receptor is expressed on cancer-associated fibroblasts, suggesting that IL-1β blockade may modulate the activity of these immunosuppressive cells (37, 39–41). In CANOPY-N, canakinumab treatment led to a reduction in the levels of regulatory T cells and CD163^+^ cells, which typically inhibit the recruitment of immune effector cells and promote an immunosuppressive TME (42), indicating that canakinumab may favor immune activation in the TME. In CANOPY-N, in contrast to preclinical models, canakinumab treatment alone did not lead to an increase in CD8^+^ T cells in the TME. In line with preclinical findings (37), decreases in the levels of neutrophils (CD66b^+^CD11b^+^ cells) were observed in most patients in CANOPY-N who received canakinumab, although this effect was also observed following pembrolizumab treatment. Noteworthy, as CD66b expression is typical of, but not exclusive to, neutrophils, other cell types that also express this antigen may have been accounted for when analyzing our samples. Taken together, our results suggest that canakinumab can modulate the NSCLC TME by decreasing immunosuppressive cell populations, and it may shift the TME toward an immune-activated status in the presence of pembrolizumab. In this context, whether patients whose tumors present immunosuppressive TME features benefit from the addition of canakinumab to an ICI-based treatment should be further investigated.

In the surgical specimens from patients from CANOPY-N who had low baseline CD8^+^ T-cell infiltration, the levels of viable tumor cells were numerically lower among those treated with the combination of canakinumab and pembrolizumab in comparison with those who received canakinumab alone, suggesting the combination may be more active in this subgroup of patients. Noteworthy, patients who had low baseline CD8^+^ T-cell infiltration and received pembrolizumab alone also had lower levels of viable tumor cells in their specimens when compared with those who received canakinumab alone, raising questions on the potential synergistic antitumor effect generated by the addition of canakinumab in these patients. Nevertheless, the three correlative analyses concerning CD8^+^ T-cell subgroups performed in CANOPY-1 samples (total CD8 count in tumor samples, T-cell phenotype, and inflamed T-cell gene signature) showed a trend for canakinumab benefit in patients with features indicative of reduced CD8^+^ T-cell infiltration, supporting the hypothesis that patients whose tumors have a low CD8^+^ T-cell infiltration may benefit from the addition of IL-1β inhibition to ICIs.

In addition to T-cell infiltration, the expression of PD-L1 is associated with responsiveness to ICI-based treatment in patients with advanced NSCLC (43). Patients with PD-L1–negative tumors derive modest benefit from ICI-based treatment and have a worse prognosis when compared with those with PD-L1–positive tumors (44, 45). Furthermore, when evaluating both PD-L1 and CD8^+^ T-cell infiltration to assess the response to ICIs, patients with both low PD-L1 and low CD8^+^ T-cell infiltration have the poorest response to ICI-based treatment (7–9). In the CANOPY-1 study, in the subgroup of patients with low CD8^+^ T-cell infiltration, we observed a PFS benefit favoring the canakinumab arm across all PD-L1 levels, suggesting that a potential effect of IL-1β blockade in this population occurs regardless of PD-L1 expression.

Our results should be interpreted in the context of the limitations present in this study. First, the exploratory nature of these analyses from two prospective studies has to be acknowledged. Second, not all patients from CANOPY-1 and CANOPY-N had evaluable samples, although patients from CANOPY-1 included in the present work were representative of the full analysis set population. Third, in CANOPY-N, the number of patients in the pembrolizumab arm was smaller than in the other two arms; hence, any comparison between the pembrolizumab and combination arms should be interpreted with caution. Fourth, on-treatment and postprogression samples from patients in CANOPY-1 were not available, which would have enabled us to further explore the dynamics of the TME during canakinumab treatment. Fifth, as the gene expression analyses were performed on whole-tumor samples (“bulk”), the observed expression profiles may have been influenced by stromal cells. In this context, single-cell and spatial transcriptomics would be an interesting next step to depict the gene expression profiles of the different TME components in response to canakinumab, distinguishing tumor cells from immune effector cells. Sixth, although we observed that low CD8 levels may identify subsets of patients who may benefit from IL-1β blockade, whether a composite biomarker, combining IHC features, transcriptomics, and potentially clinical characteristics, may be a more accurate predictor of canakinumab benefit, should be further investigated. Despite the aforementioned limitations, to our knowledge, the present is the first work to characterize and report the effects of IL-1β blockade in the TMEs of patients with NSCLC. Furthermore, the fact that all analyses were conducted using datasets from two prospective, randomized studies strengthens our findings.

In conclusion, our study indicates that IL-1β blockade in combination with an ICI may modulate the NSCLC TME and shift it toward an immune-activated status. Our findings suggest that IL-1β blockade may benefit a subset of patients with immunosuppressive TME features who typically derive little benefit from ICIs. Prospective validation of these findings is warranted, and further work is needed to identify patients who may benefit from the addition of IL-1β inhibition to ICI-based treatment.

## Supplementary Material

Supplementary Data tables Tables S1 - S4

Figure S1 A, CANOPY-1 and B, CANOPY-N study designs.

Figure S2 T-cell phenotypes derived from VisioPharm IA software algorithm were established under pathology review using panCK (purple) as the tumor marked-up area. Semi-automatic scoring of CD8 (brown) in panCK-negative tumor stroma and panCK-positive carcinoma cell compartments was performed, and representative T-cell phenotypes are depicted.

Figure S3 A, Classification of T-cell phenotypes based on CD8 IHC. In samples with paired RNA-Seq data, B, T-cell–inflamed gene signature and C, fibroblast gene signatures, by T-cell phenotype.

Figure S4 Cell phenotypes assessed by QIF: A, CD11b/CD66b stained for polymorphonuclear granulocytes (or neutrophils); B, CD163 stained for monocyte-lineage macrophages (or tumor-associated macrophages); and C, FOXP3/CD3 stained for regulatory T cells.

Figure S5 Comparison of CD8 subgroups (median cut-off) with T-cell phenotypes for CANOPY-1, by A, CD8 level and B, T-cell phenotype.

Figure S6 A, PFS and B, OS by low T-cell–inflamed signature and C, PFS and D, OS by high T-cell–inflamed signature for CANOPY-1.

Figure S7 A, CD8 central tumor levels by PD-L1 subgroups and B, summary of patient characteristics at baseline by PD-L1 subgroup for CANOPY-1.

Figure S8 Principal component analysis to deconvolve independent TME features in CANOPY-1. Dimension 1 indicates total immune infiltration and dimension 2 indicates the balance between immune supressive signatures and antitumor immunity.

Figure S9 Distribution of T-cell subgroups in CANOPY-1 using A, dimension 1, total immune infiltration and B, dimension 2, balance between immune suppressive signatures and antitumor immunity.

Figure S10 Changes in T-cell phenotype distribution from screening to surgery for CANOPY-N as A, a T-cell phenotype count and B, a fraction of the total phenotypes per arm/time point.

Figure S11 Distribution of viable tumor cells in CANOPY-N surgery samples in A, all patients, B, the CD8-low subgroup, and C, the CD8-high subgroup.
