# Supplementary material for: The Impact of the Tumor Microenvironment on the Effect of IL-1β Blockade in NSCLC: Biomarker Analyses from CANOPY-1 and CANOPY-N Trials
Source: Cancer Res Commun. 2025 Apr 18;5(4):632–46. doi: 10.1158/2767-9764.CRC-24-0490 (PMC12006968; doi:10.1158/2767-9764.CRC-24-0490)
Supplement: Supplementary Data tables — Tables S1 - S4 [file crc-24-0490_supplementary_data_tables_suppst1-st4.docx]

**Supplementary Tables**

**Supplementary Table S1. Gene signatures**

|  | Reference | Genes |
| --- | --- | --- |
| Fibroblast | Novartis internal signature | *GXYLT2, COL3A1, ADAMTS12, POSTN, CCN4, COL5A2, COLEC12, BNC2, COL6A3, MFAP5, FAP, FNDC1, ITGA11, COL8A1, THBS2, COL10A1, VGLL3, FBLN2, KCNE4, SFRP2, MSC-AS1, LGI2, ASPN, SULF1, TNN, CTHRC1, MEDAG, C3orf80, CPXM1, CLMP, MMP2, GREM1, TNFAIP6, HAS2, GPC6, EMILIN1, RGS4, DKK2, EDNRA, ALPK2, LRRC15, COL1A1, COL1A2* |
| IFNG_10gene | <https://www.ncbi.nlm.nih.gov/pmc/articles/PMC5531419/> | *IFNG, IDO1, CXCL11, CXCL10, PRF1, CCR5, HLA-DRA, STAT1, GZMA, CXCL9* |
| IL1B_extended | Novartis internal signature | *IL1B, IL1R1, IL1RN, IL1R2, IL6, IL18BP, IL18, CXCL8* |
| IL1b_macrophage | <https://aacrjournals.org/cancerimmunolres/article/11/6/777/726528> | *IL1B, CXCL1, CXCL2, CXCL3, IL6, NLRP3* |
| Immune_Chemokine_neutrophil | <https://www.nature.com/articles/s41591-020-1082-2> | *CXCL5, CXCL1, CXCL2, CXCL3, CXCL8, CCL20* |
| Immune_TypeI_IFN | <https://www.nature.com/articles/s41591-020-1082-2> | *RSAD2, ISG15, IFIH1, IFIT2, IFIT1, OAS2, BST2, USP18, IFI6, SP100, IFI44L, MX1, MX2, OASL* |
| ISG_IFN1 | <https://www.nature.com/articles/s41591-018-0302-5> | *TDRD7, IFIH1, PNPT1, USP18, SAMD9L, NMI, RSAD2, TRIM25, TRAFD1, DHX58, UBE2L6, EPSTI1, GBP4, ISG15, CXCL10, BST2, OGFR, DDX60, OASL, CASP1, CMPK2, TRIM21, IRF7, ISG20, PSME2, HERC6, PARP14, TRIM14, SP110, IFI35, IFIT2, EIF2AK2, ADAR, RTP4, IFIT3, STAT2, MX1, PARP12* |
| Lineage_macrophage | <https://www.sciencedirect.com/science/article/pii/S0092867418311784?via%3Dihub> | *FCGR1A, CD14, ITGAL, ITGAM, ENG, ITGAX, CD68, CD80, CD86, LILRB4, LAMP2, CD33, CCR5, CD163, TLR2, TLR4, CSF1R, FUT4* |
| Lineage_neutrophil | <https://jitc.biomedcentral.com/articles/10.1186/s40425-017-0215-8> | *CSF3R, FCGR3B, SIGLEC5, S100A12, FCAR, FPR1, CEACAM3* |
| M2 | <https://ascopubs.org/doi/abs/10.1200/JCO.2018.36.15_suppl.3020> | *TGFB1 ,MSR1, PPBP, IL1A, CD14, IL1RN, S100A8, MRC1, S100A9, S100A12, PF4, C5AR1, SAA1, CD163, CXCL8, CXCR1, CXCR2, ARG1* |
| Suppressive_TME | Novartis internal signature | *TGFB1, IL10, TGFB2, CCL17, ARG1* |
| Tcell_inflamed_18gene | <https://science.sciencemag.org/content/362/6411/eaar3593.editor-summary> | *TIGIT, PSMB10, IDO1, CD274, CXCR6, CD27, HLA-DQA1, CD8A, CCL5, NKG7, HLA-DRB1, STAT1, HLA-E, CMKLR1, CXCL9, PDCD1LG2, CD276, LAG3* |
| TGFb | <https://jitc.bmj.com/content/11/11/e007353.long> | *FOXS1, SOX4, PMEPA1, HEYL, FAP, ALOX5AP, COL1A1, TBC1D2B* |
| TIS_14gene | Novartis internal signature | *CCL5, CTSW, CD8B, GZMA, XCL2, KLRC1, CD8A, KLRK1, GNLY, PRF1, GZMB, GZMH, LAG3, KLRD1* |
| Angiogenesis | Novartis internal signature | *AKT1, AKT2, AKT3, BCL10, CARD11, CAV1, CDH5, CHUK, CTSB, CXCR1, CXCR2EGFR, ELK1, FASN, FNTA, FOS, GNAI1, GNAI2, GNAI3, GNAO1, GNAZ, GNB1, GNB2, GNB3, GNB4, GNB5, GNG10, GNG11, GNG12, GNG13, GNG2, GNG3, GNG4, GNG5, GNG7, GNG8, GNGT1, GNGT2, HBEGF, HMGCR, IKBKB, IKBKG, CXCL8, JAK2, KDR, LDLR, MALT1, MAP2K1, MAPK1, MAPK3, MBTPS1, MBTPS2, MMP14, NFKB1, NFKB2, NFKBIA, NFKBIB, NFKBIE, PAK1, PDPK1, PGGT1B, PIK3CA, PIK3CB, PIK3CD, PIK3CG, PIK3R1, PIK3R2, PIK3R5, PTK2, PXN, RAC1, REL, RELA, RELB, RHOA, SCAP, SRC, SREBF1, SREBF2, STAT3, UBB, UBC, UBE2N, VEGFA* |

**Supplementary Table S2. CANOPY-1 and CANOPY-N samples with available biomarker results**

**CANOPY-1**

| **Biomarker, *n*** | **Both arms (*n* = 643)** | **Placebo (*n* = 323)** | **Canakinumab (*n* = 320)** |
| --- | --- | --- | --- |
| **PD-L1^a^** | 643 | 323 | 320 |
| **CD8/panCK^b^** | 500^c^ | 252 | 248 |
| **RNA-Seq^b^** | 298 | 146 | 152 |

**CANOPY-N**

| **Biomarker, *n*** | **Arm** | **Screening** | **Surgery** | **Paired** |
| --- | --- | --- | --- | --- |
| **CD8/panCK^b^** | Canakinumab | 30 | 26 | 23 |
|  | Canakinumab + pembrolizumab | 32 | 28 | 26 |
|  | Pembrolizumab | 11 | 13 | 8 |
| **CD3, CD11b, CD19, CD66b, CD163, CD56^b^** | Canakinumab | 29 | 29 | 27 |
|  | Canakinumab + pembrolizumab | 27 | 26 | 22 |
|  | Pembrolizumab | 13 | 12 | 11 |
| **CD3, FOXP3, granzyme B, cytokeratin^b^** | Canakinumab | 13 | 13 | 13 |
|  | Canakinumab + pembrolizumab | 9 | 10 | 9 |
|  | Pembrolizumab | 6 | 6 | 6 |

^a^Central PD-L1 was a stratification factor (all patients tested).
^b^Analysis performed for patients with sufficient biopsy, for both quality and quantity.
^c^T-cell phenotype could be determined in 499 out of the 500 samples: one sample did not have valid stroma:tumor ratio and, therefore, was classified as phenotype “unknown”.

**Supplementary Table S3. Interaction test of CD8 subgroup and treatment arm for OS and PFS**

|  | **Coef** | **Exp. coef** | **SE coef** | ***P*-value** |
| --- | --- | --- | --- | --- |
| **OS** |  |  |  |  |
| **Canakinumab + pembrolizumab + CTx^a^: Excluded^b^** | 0.35 | 1.42 | 0.35 | 0.31 |
| **Canakinumab + pembrolizumab + CTx^a^: Inflamed^b^** | 0.05 | 1.05 | 0.42 | 0.90 |
| **Canakinumab + pembrolizumab + CTx^a^: Above the median cut-off (high)^c^** | 0.07 | 1.07 | 0.26 | 0.79 |
| **PFS** |  |  |  |  |
| **Canakinumab + pembrolizumab + CTx^a^: Excluded^b^** | 0.25 | 1.28 | 0.30 | 0.41 |
| **Canakinumab + pembrolizumab + CTx^a^: Inflamed^b^** | 0.03 | 1.04 | 0.35 | 0.92 |
| **Canakinumab + pembrolizumab + CTx^a^: Above the median cut-off (high)^c^** | 0.29 | 1.34 | 0.22 | 0.18 |

Reference level of the treatment arm: ^a^Placebo + pembrolizumab + CTx.

Reference level of CD8 subgroup: ^b^CD8^+^ desert; ^c^CD8-low.

**Abbreviations:** Coef, coefficient; CTx, chemotherapy treatment; Exp, exponential; SE, standard error

**Supplementary Table S4. Representativeness of study participants**

| Cancer type(s)/subtype(s)/stage(s)/condition | Stage IB-IIIA and Stage IIIB-IV NSCLC |
| --- | --- |
| Considerations related to: |  |
| Sex | The incidence of lung cancer, of which NSCLC makes up ~85% (1), is higher in males than females (53% vs 47%); this gap has been closing in recent years (2). |
| Age | Lung cancer mainly occurs in older adults with most people diagnosed being 65 years or age or older (1). |
| Race/ethnicity | In the USA, the American Cancer Society states that Black men are about 12% more likely to develop lung cancer than White men. The rate is about 16% lower in Black women than in White women (3).  This conflicts with data from US Centers for Disease Control and Prevention (CDC), that suggests the rate of lung cancer is highest in White people, with an incidence rate of around 53.2 per 100,000 people, respectively. American Indian people and Alaska Native people have the next highest rate at 51.8 per 100,000 followed by Black people with a rate of 50.7 per 100,000. The rates for lung cancer among Asian and Pacific Islanders and Hispanic people are 31.0 and 26.4 per 100,000 people, respectively (4). |
| Geography | In males, the highest incidence rates for lung cancer are observed in Micronesia/Polynesia, Eastern and Southern Europe, Eastern Asia, and Western Asia, where Turkey has the highest rate among men globally. Incidence rates remain generally low in Africa, although they range from intermediate to high in both Southern and Northern regions. Among women, the highest incidence rates are in Northern America, Northern and Western Europe, Micronesia/Polynesia, and Australia/New Zealand, with Hungary having the highest country-specific rates (5). |
| Other considerations | International variation in lung cancer rates and trends largely reflects the maturity of the tobacco epidemic; this pattern may well change as the tobacco epidemic evolves given that 80% of smokers aged ≥15 years resided in low-income and middle-income countries in 2016 (5). |
| Overall representativeness of patients selected for this study | In the subset of patients selected from CANOPY-1 and CANOPY-N trials for this study, patients were slightly younger and there was a higher proportion of males than in the general population of patients with lung cancer. Representation of Black patients was low with most patients being White or Asian; however, proportions may be more representative of the race/ethnicity composition of countries with higher incidence of lung cancer   - 45% were aged ≥65 years - 70% were male - 82% were former or current smokers - 58% were White, 33% were Asian, 1% were Black and 8% were unknown   For these two trials, patients were recruited across North and South America, Western and Eastern Europe, Australia, and Asia. These sites are aligned with those where lung cancer incidence is high in the general population. |

**References**

1. Ganti AK, Klein AB, Cotarla I, Seal B, Chou E. Update of Incidence, Prevalence, Survival, and Initial Treatment in Patients With Non-Small Cell Lung Cancer in the US. JAMA Oncol **2021**;7:1824-1832 doi: 10.1001/jamaoncol.2021.4932.
2. Fu Y, Liu J, Chen Y, Liu Z, Xia H, Xu H. Gender Disparities in Lung Cancer Incidence in the United States During 2001-2019. Sci Rep **2023**;13:12581 doi: 10.1038/s41598-023-39440-8.
3. American Cancer Society. Key Statistics for Lung Cancer. Available at: <https://www.cancer.org/cancer/types/lung-cancer/about/key-statistics.html>. Accessed March 2025.
4. U.S. Centers for Disease Control and Prevention. United States Cancer Statistics: Data Visualizations. Available at: <https://gis.cdc.gov/Cancer/USCS/#/Demographics/>. Accessed March 2025.
5. Bray F, Laversanne M, Sung H, Ferlay J, Siegel R, Soerjomataram I, *et al.* Global Cancer Statistics 2022: GLOBOCAN Estimates of Incidence and Mortality Worldwide for 36 Cancers in 185 Countries. CA Cancer J Clin **2024**;74:229-263 doi: 10.3322/caac.21834.
