# Supplementary material for: The Impact of the Tumor Microenvironment on the Effect of IL-1β Blockade in NSCLC: Biomarker Analyses from CANOPY-1 and CANOPY-N Trials
Source: Cancer Res Commun. 2025 Apr 18;5(4):632–46. doi: 10.1158/2767-9764.CRC-24-0490 (PMC12006968; doi:10.1158/2767-9764.CRC-24-0490)
Supplement: Figure S1 — A, CANOPY-1 and B, CANOPY-N study designs. [file crc-24-0490_figure_s1_suppsf1.pdf]

Supplementary Figure S1. A, CANOPY-1 and **B,** CANOPY-N study designs.

A.

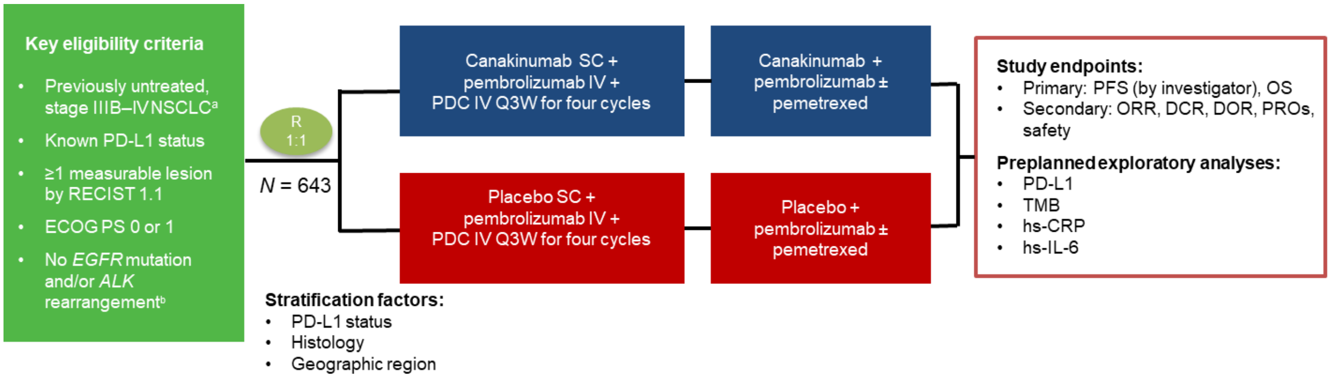

B.

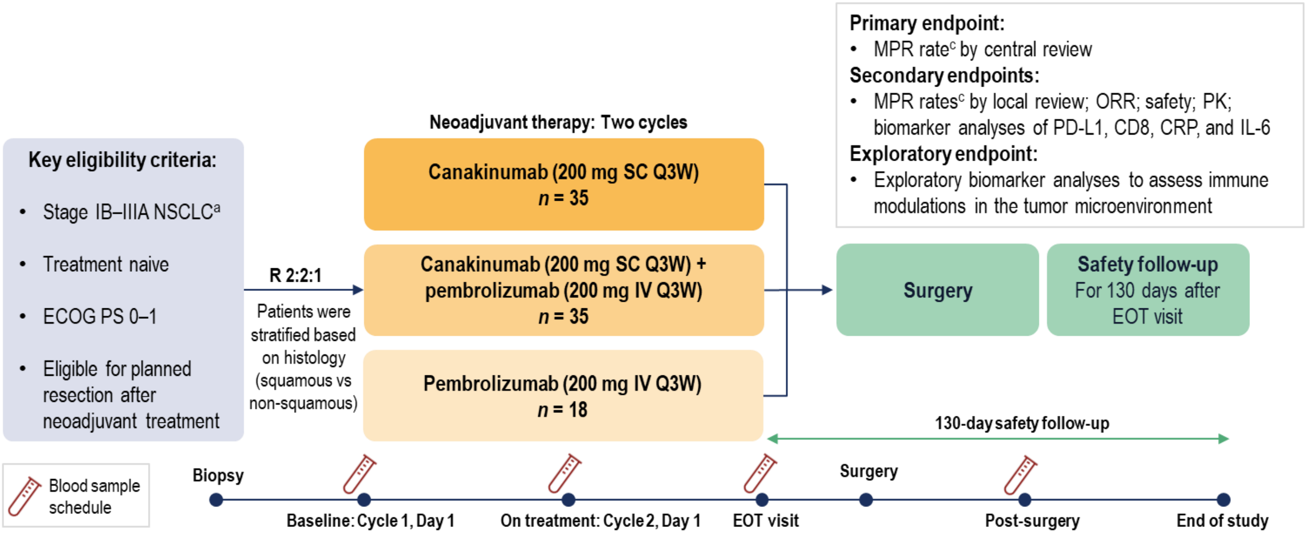

[a]Stages were defined using the AJCC/UICC version 8 staging recommendations. [b]Patients with known *ROS1* rearrangement or *BRAF* V600 mutation were excluded if required by local guidelines. [c]MPR rate: percentage of patients with ≤10% residual viable cancer cells on surgical sample.

Abbreviations: AJCC, American Joint Committee on Cancer; CRP, C-reactive protein; DCR, disease control rate; DOR, duration of response; ECOG, Eastern Cooperative Oncology Group; EOT, end of treatment; hs, high sensitivity; IV, intravenously; ORR, overall response rate; PDC, platinum doublet chemotherapy; PRO, patient-reported outcome; PS, performance status; RECIST 1.1, Response Evaluation Criteria In Solid Tumors 1.1; TMB, tumor mutational burden; Q3W, once every 3 weeks; Q4W, once every 4 weeks; R, randomized; SC, subcutaneously; UICC, Union for International Cancer Control.
