# Supplementary material for: The Impact of the Tumor Microenvironment on the Effect of IL-1β Blockade in NSCLC: Biomarker Analyses from CANOPY-1 and CANOPY-N Trials
Source: Cancer Res Commun. 2025 Apr 18;5(4):632–46. doi: 10.1158/2767-9764.CRC-24-0490 (PMC12006968; doi:10.1158/2767-9764.CRC-24-0490)
Supplement: Figure S2 — T-cell phenotypes derived from VisioPharm IA software algorithm were established under pathology review using panCK (purple) as the tumor marked-up area. Semi-automatic scoring of CD8 (brown) in panCK-negative tumor stroma and panCK-positive carcinoma cell compartments was performed, and representative T-cell phenotypes are depicted. [file crc-24-0490_figure_s2_suppsf2.pdf]

Supplementary Figure S2. T-cell phenotypes derived from VisioPharm IA software algorithm were established under pathology review using panCK (purple) as the tumor marked-up area. Semi-automatic scoring of CD8 (brown) in panCK-negative tumor stroma and panCK-positive carcinoma cell compartments was performed, and representative T-cell phenotypes are depicted.

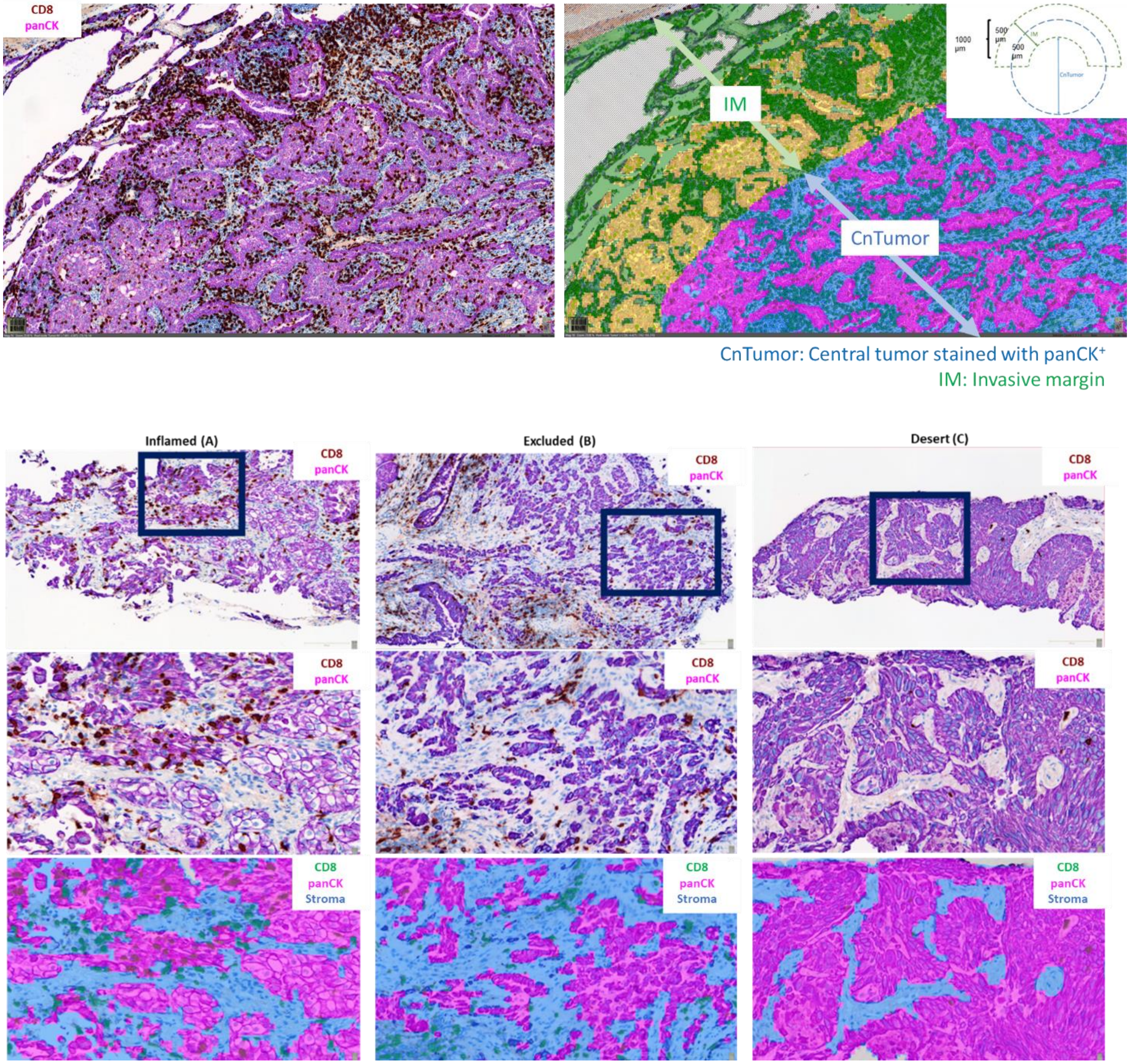

Abbreviations: CnTumor; central tumor stained with panCK^{+}; IM, invasive margin.
