# Supplementary material for: The Impact of the Tumor Microenvironment on the Effect of IL-1β Blockade in NSCLC: Biomarker Analyses from CANOPY-1 and CANOPY-N Trials
Source: Cancer Res Commun. 2025 Apr 18;5(4):632–46. doi: 10.1158/2767-9764.CRC-24-0490 (PMC12006968; doi:10.1158/2767-9764.CRC-24-0490)

Supplementary Figure S3. A, Classification of T-cell phenotypes based on CD8 IHC. In samples with paired RNA-Seq data, **B,** T-cell–inflamed gene signature and **C,** fibroblast gene signatures, by T-cell phenotype.

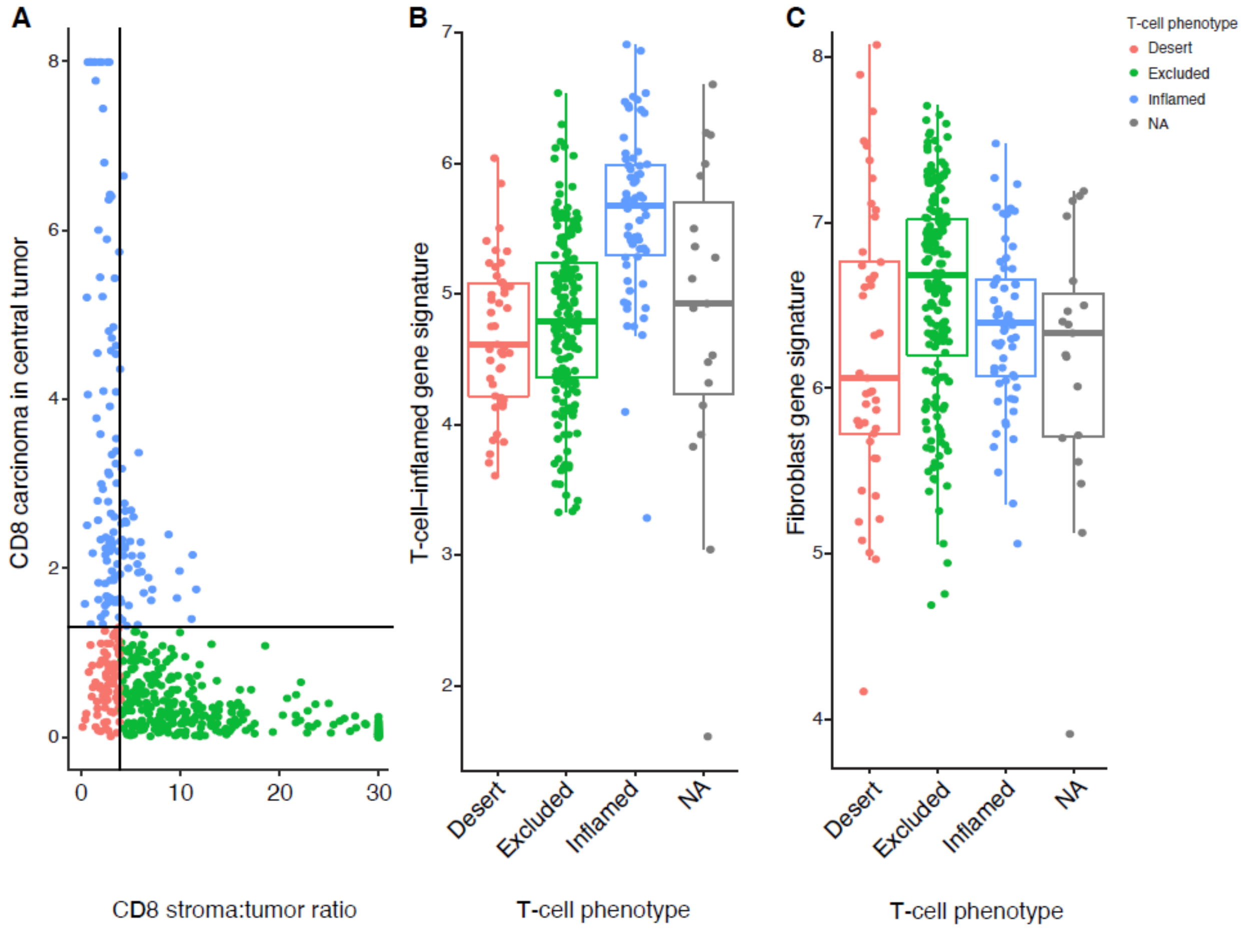

Supplement: Figure S3 — A, Classification of T-cell phenotypes based on CD8 IHC. In samples with paired RNA-Seq data, B, T-cell–inflamed gene signature and C, fibroblast gene signatures, by T-cell phenotype. [file crc-24-0490_figure_s3_suppsf3.pdf]
