# Supplementary material for: The Impact of the Tumor Microenvironment on the Effect of IL-1β Blockade in NSCLC: Biomarker Analyses from CANOPY-1 and CANOPY-N Trials
Source: Cancer Res Commun. 2025 Apr 18;5(4):632–46. doi: 10.1158/2767-9764.CRC-24-0490 (PMC12006968; doi:10.1158/2767-9764.CRC-24-0490)
Supplement: Figure S4 — Cell phenotypes assessed by QIF: A, CD11b/CD66b stained for polymorphonuclear granulocytes (or neutrophils); B, CD163 stained for monocyte-lineage macrophages (or tumor-associated macrophages); and C, FOXP3/CD3 stained for regulatory T cells. [file crc-24-0490_figure_s4_suppsf4.pdf]

Supplementary Figure S4. Cell phenotypes assessed by QIF: **A,** CD11b/CD66b stained for polymorphonuclear granulocytes (or neutrophils); **B,** CD163 stained for monocyte-lineage macrophages (or tumor-associated macrophages); and **C,** FOXP3/CD3 stained for regulatory T cells.

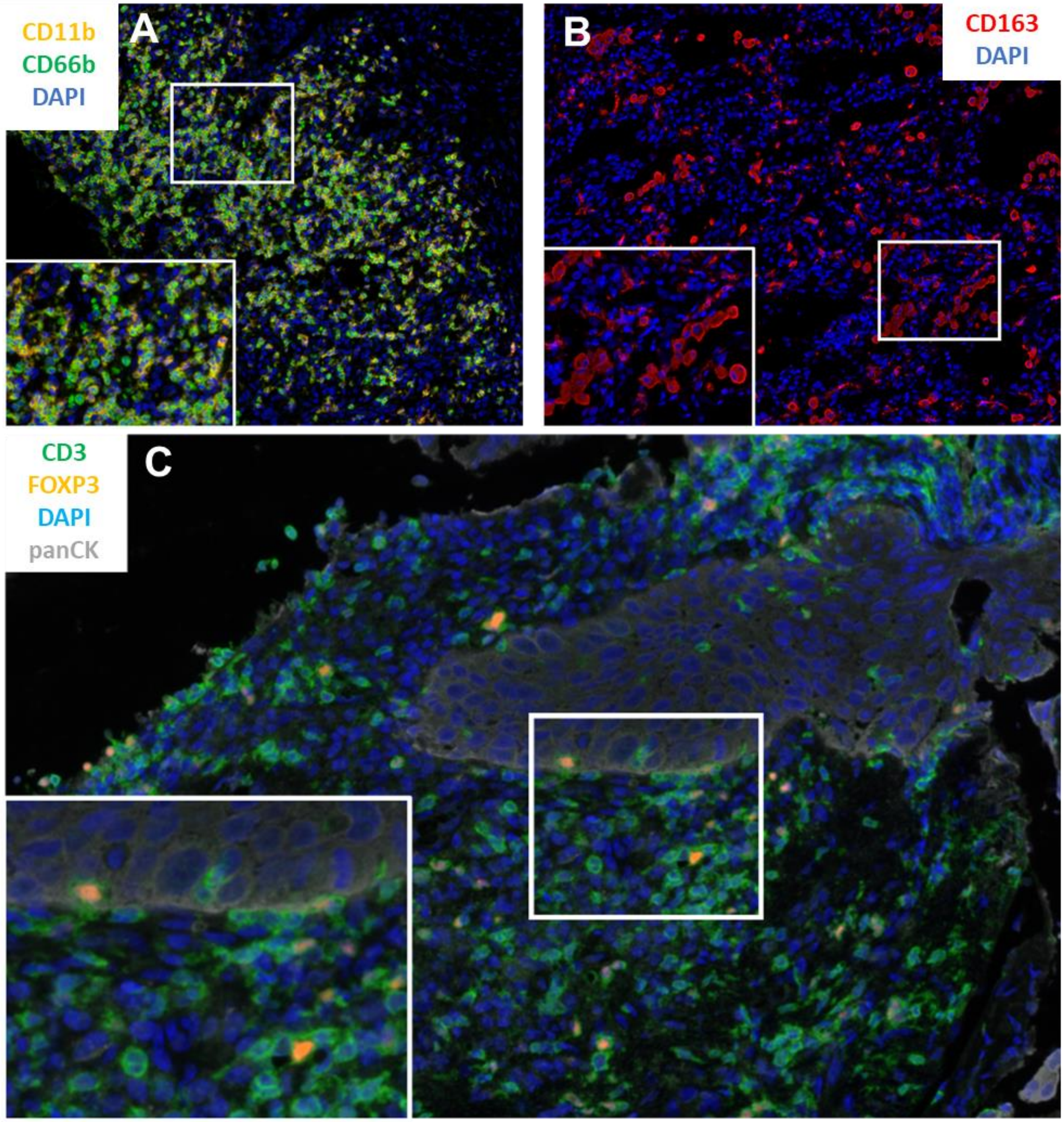

Abbreviations: DAPI, 4′,6-diamidino-2-phenylindole; QIF, quantitative immunofluorescence.
