# Supplementary material for: The Impact of the Tumor Microenvironment on the Effect of IL-1β Blockade in NSCLC: Biomarker Analyses from CANOPY-1 and CANOPY-N Trials
Source: Cancer Res Commun. 2025 Apr 18;5(4):632–46. doi: 10.1158/2767-9764.CRC-24-0490 (PMC12006968; doi:10.1158/2767-9764.CRC-24-0490)

Supplementary Figure S5. Comparison of CD8 subgroups (median cut-off) with T-cell phenotypes for CANOPY-1, by **A,** CD8 level and **B,** T-cell phenotype.

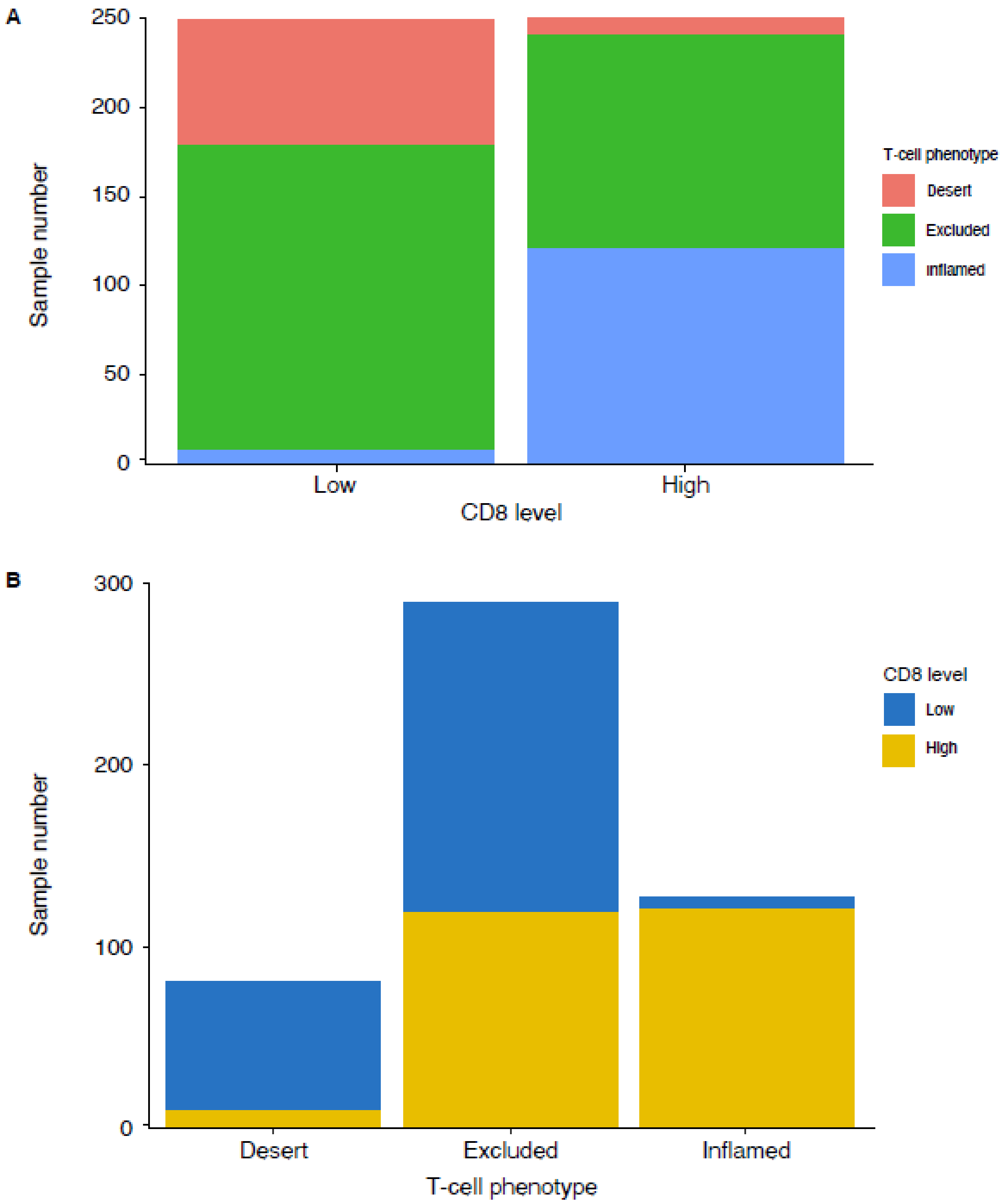

Supplement: Figure S5 — Comparison of CD8 subgroups (median cut-off) with T-cell phenotypes for CANOPY-1, by A, CD8 level and B, T-cell phenotype. [file crc-24-0490_figure_s5_suppsf5.pdf]
