# Supplementary material for: The Impact of the Tumor Microenvironment on the Effect of IL-1β Blockade in NSCLC: Biomarker Analyses from CANOPY-1 and CANOPY-N Trials
Source: Cancer Res Commun. 2025 Apr 18;5(4):632–46. doi: 10.1158/2767-9764.CRC-24-0490 (PMC12006968; doi:10.1158/2767-9764.CRC-24-0490)
Supplement: Figure S6 — A, PFS and B, OS by low T-cell–inflamed signature and C, PFS and D, OS by high T-cell–inflamed signature for CANOPY-1. [file crc-24-0490_figure_s6_suppsf6.pdf]

Supplementary Figure S6. A, PFS and **B,** OS by low T-cell–inflamed signature and **C,** PFS and **D**, OS by high T-cell–inflamed signature for CANOPY-1.

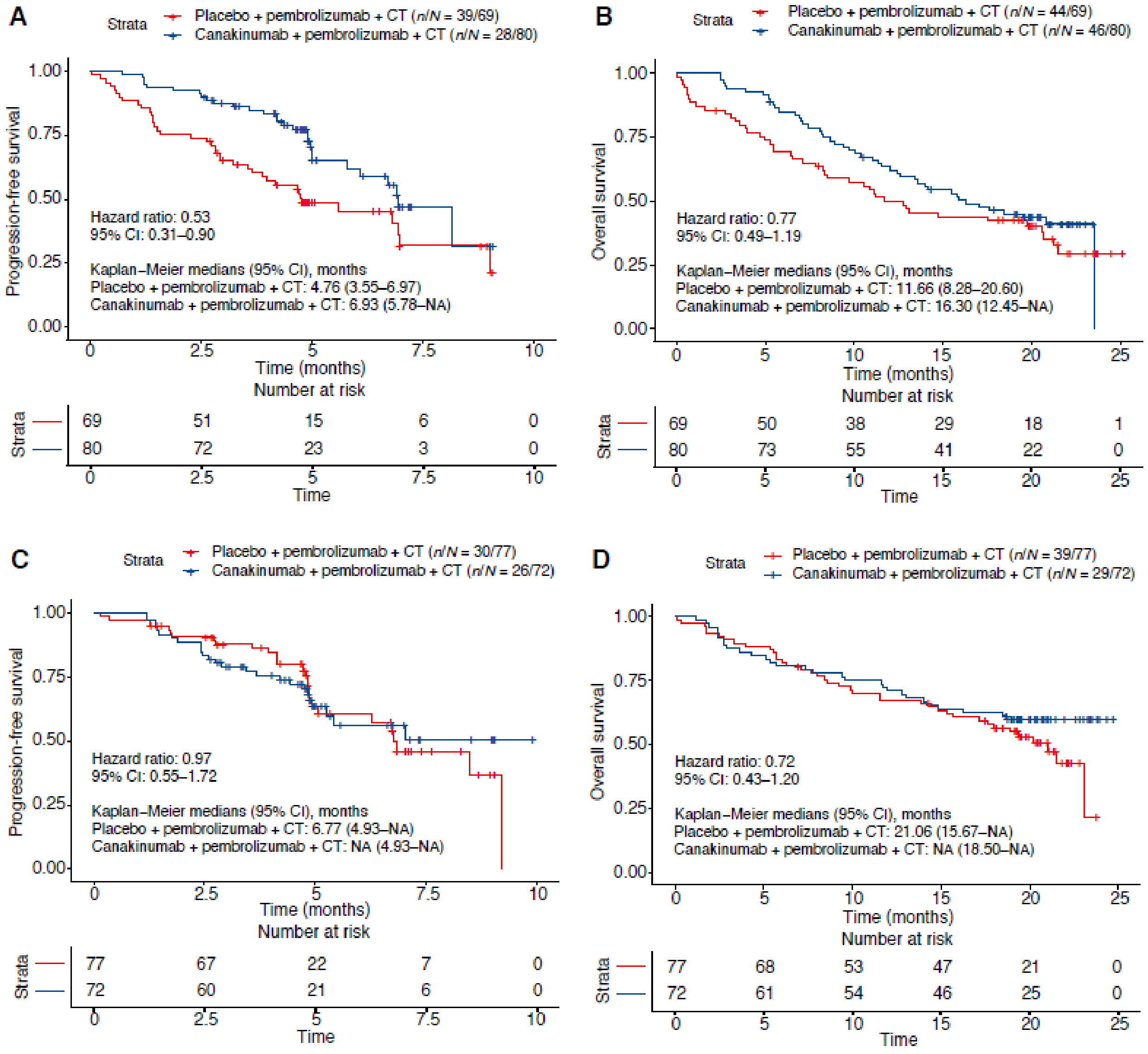

Abbreviations: CT, chemotherapy.
