# Supplementary material for: The Impact of the Tumor Microenvironment on the Effect of IL-1β Blockade in NSCLC: Biomarker Analyses from CANOPY-1 and CANOPY-N Trials
Source: Cancer Res Commun. 2025 Apr 18;5(4):632–46. doi: 10.1158/2767-9764.CRC-24-0490 (PMC12006968; doi:10.1158/2767-9764.CRC-24-0490)
Supplement: Figure S7 — A, CD8 central tumor levels by PD-L1 subgroups and B, summary of patient characteristics at baseline by PD-L1 subgroup for CANOPY-1. [file crc-24-0490_figure_s7_suppsf7.pdf]

Supplementary Figure S7. A, CD8 central tumor levels by PD-L1 subgroups and **B,** summary of patient characteristics at baseline by PD-L1 subgroup for CANOPY-1.

A

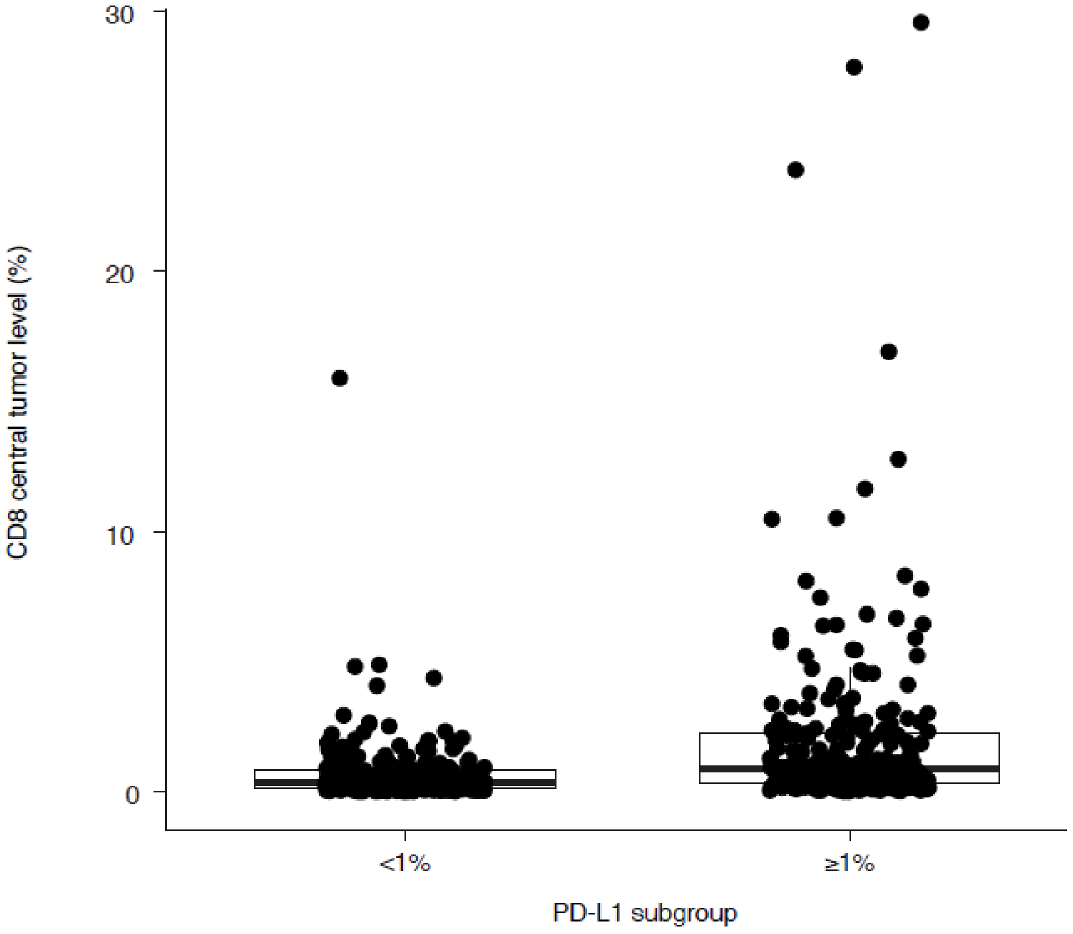

B

	PD-L1 <1% (*n* = 321)	PD-L1 ≥1% (*n* = 322)	Total (*N* = 643)
**CD8 level, n (%)**			
Number of missing	85	58	143
Low	135 (57)	115 (44)	250 (50)
High	101 (43)	149 (56)	250 (50)
