# Supplementary material for: The Impact of the Tumor Microenvironment on the Effect of IL-1β Blockade in NSCLC: Biomarker Analyses from CANOPY-1 and CANOPY-N Trials
Source: Cancer Res Commun. 2025 Apr 18;5(4):632–46. doi: 10.1158/2767-9764.CRC-24-0490 (PMC12006968; doi:10.1158/2767-9764.CRC-24-0490)
Supplement: Figure S8 — Principal component analysis to deconvolve independent TME features in CANOPY-1. Dimension 1 indicates total immune infiltration and dimension 2 indicates the balance between immune supressive signatures and antitumor immunity. [file crc-24-0490_figure_s8_suppsf8.pdf]

Supplementary Figure S8. Principal component analysis to deconvolve independent TME features in CANOPY-1. Dimension 1 indicates total immune infiltration and dimension 2 indicates the balance between immune supressive signatures and antitumor immunity.

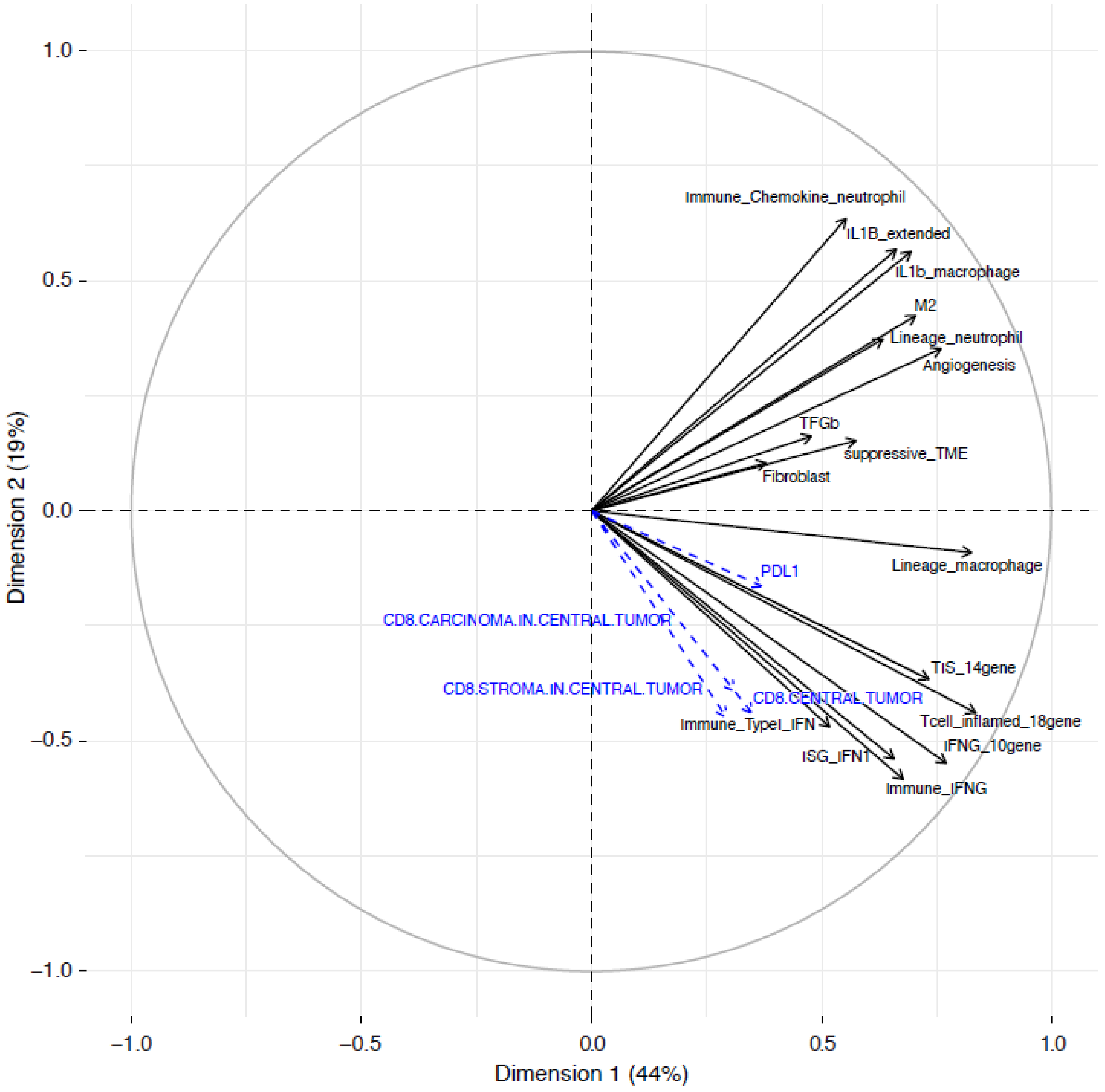

Abbreviations: IFN, interferon; TFG, transforming growth factor.
