# Supplementary material for: The Impact of the Tumor Microenvironment on the Effect of IL-1β Blockade in NSCLC: Biomarker Analyses from CANOPY-1 and CANOPY-N Trials
Source: Cancer Res Commun. 2025 Apr 18;5(4):632–46. doi: 10.1158/2767-9764.CRC-24-0490 (PMC12006968; doi:10.1158/2767-9764.CRC-24-0490)

Supplementary Figure S9. Distribution of T-cell subgroups in CANOPY-1 using **A,** dimension 1, total immune infiltration and **B,** dimension 2, balance between immune suppressive signatures and antitumor immunity.

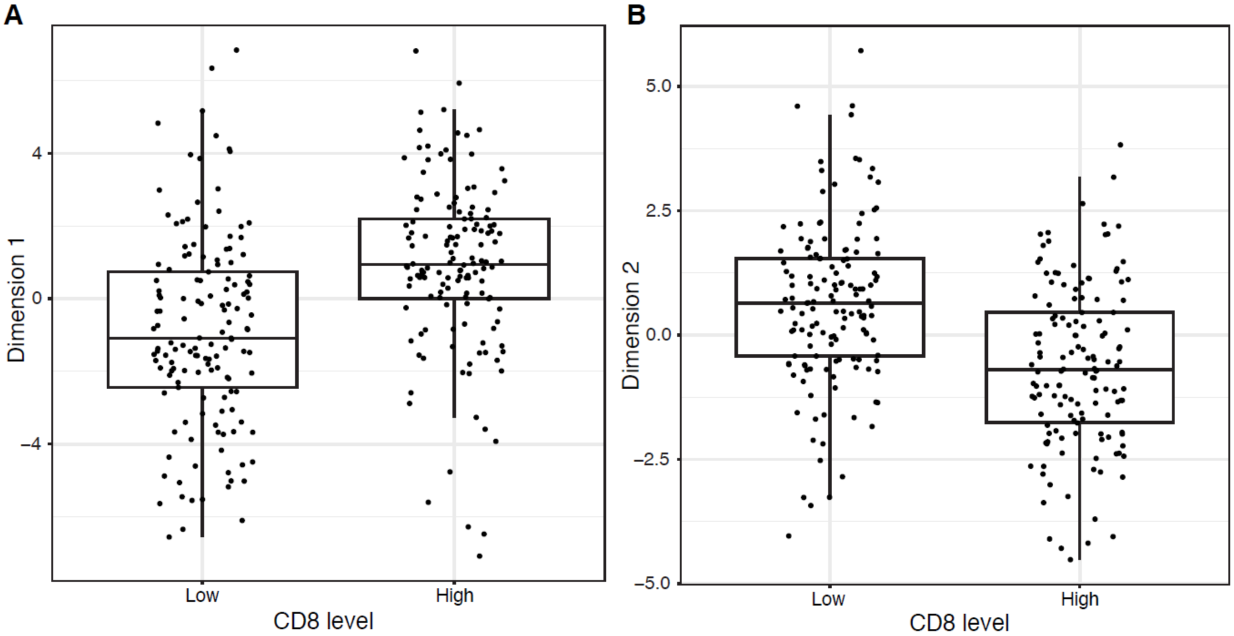

Supplement: Figure S9 — Distribution of T-cell subgroups in CANOPY-1 using A, dimension 1, total immune infiltration and B, dimension 2, balance between immune suppressive signatures and antitumor immunity. [file crc-24-0490_figure_s9_suppsf9.pdf]
