# Supplementary material for: The Impact of the Tumor Microenvironment on the Effect of IL-1β Blockade in NSCLC: Biomarker Analyses from CANOPY-1 and CANOPY-N Trials
Source: Cancer Res Commun. 2025 Apr 18;5(4):632–46. doi: 10.1158/2767-9764.CRC-24-0490 (PMC12006968; doi:10.1158/2767-9764.CRC-24-0490)

Supplementary Figure S10. Changes in T-cell phenotype distribution from screening to surgery for CANOPY-N as **A,** a T-cell phenotype count and **B,** a fraction of the total phenotypes per arm/time point.

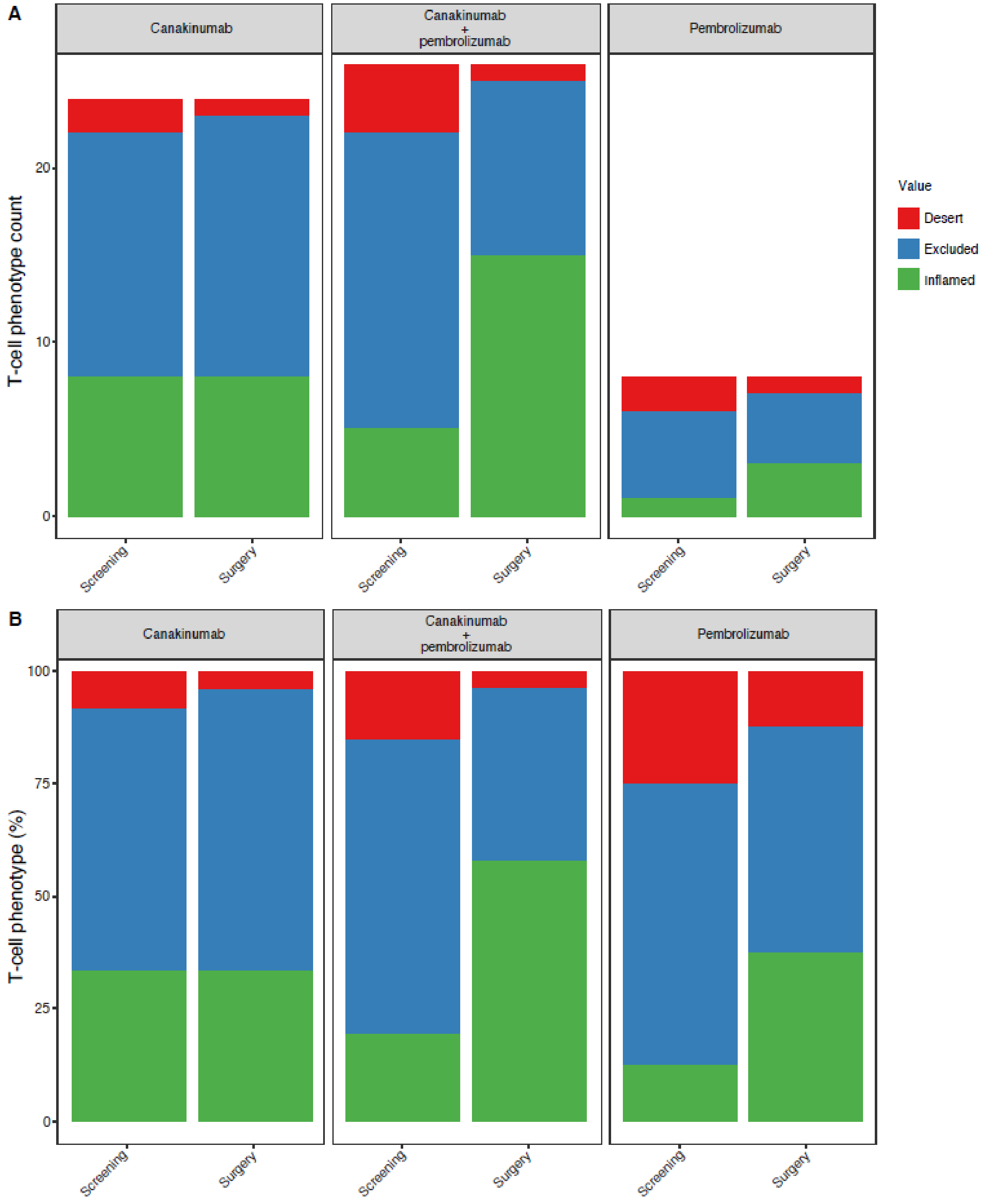

Supplement: Figure S10 — Changes in T-cell phenotype distribution from screening to surgery for CANOPY-N as A, a T-cell phenotype count and B, a fraction of the total phenotypes per arm/time point. [file crc-24-0490_figure_s10_suppsf10.pdf]
