# Supplementary material for: The Impact of the Tumor Microenvironment on the Effect of IL-1β Blockade in NSCLC: Biomarker Analyses from CANOPY-1 and CANOPY-N Trials
Source: Cancer Res Commun. 2025 Apr 18;5(4):632–46. doi: 10.1158/2767-9764.CRC-24-0490 (PMC12006968; doi:10.1158/2767-9764.CRC-24-0490)

Supplementary Figure S11. Distribution of viable tumor cells in CANOPY-N surgery samples in **A,** all patients, **B,** the CD8-low subgroup, and **C,** the CD8-high subgroup.

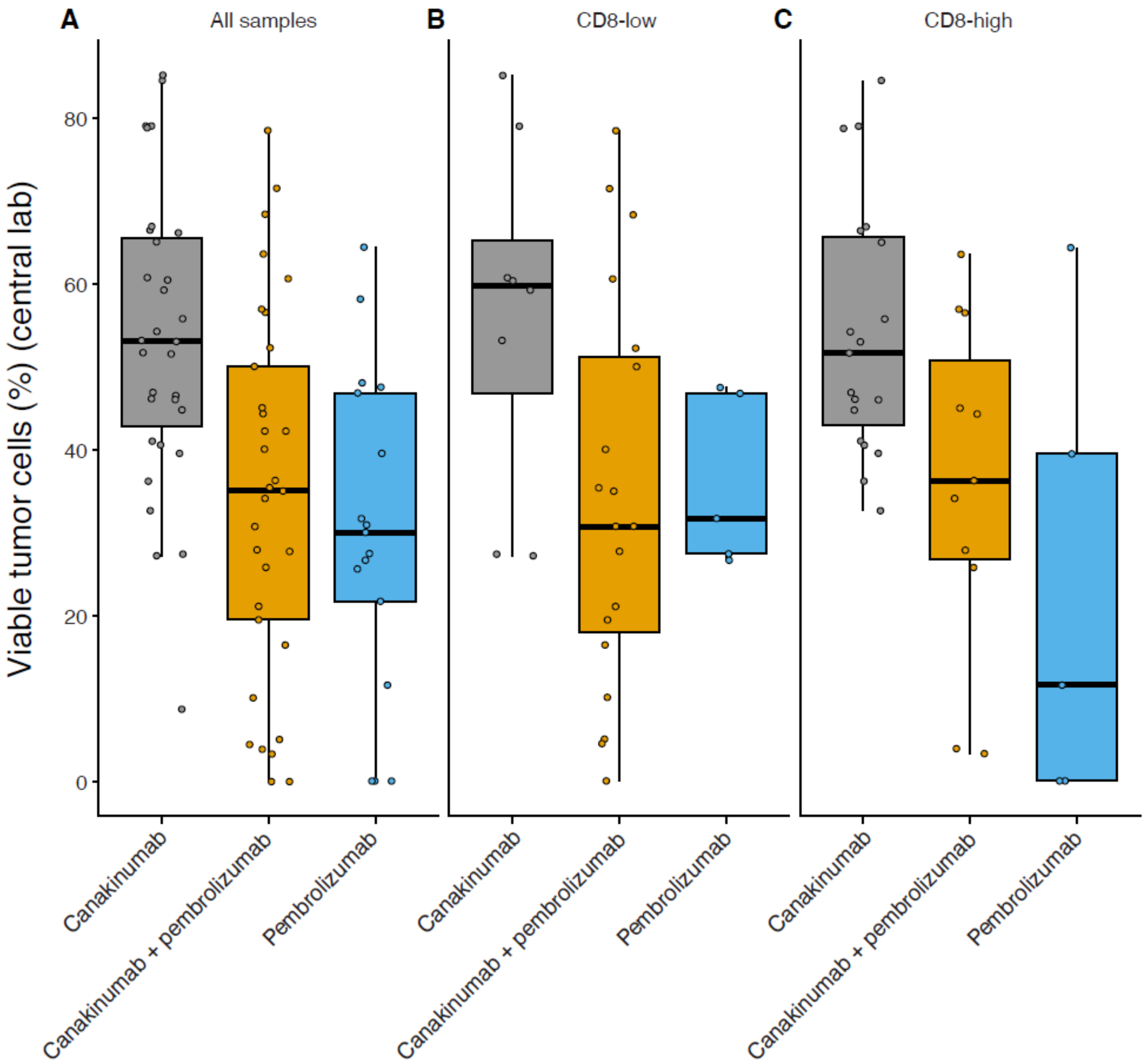

Supplement: Figure S11 — Distribution of viable tumor cells in CANOPY-N surgery samples in A, all patients, B, the CD8-low subgroup, and C, the CD8-high subgroup. [file crc-24-0490_figure_s11_suppsf11.pdf]
